# Influence of methyl substitution on linear diboronic acids: toward spiroborate covalent organic framework formation in *N*,*N*-diethylformamide†

**DOI:** 10.1039/d5ta02297e

**Published:** 2025-05-21

**Authors:** Xue Wang, Qiang Zhu, Hang Qu, Xiang Zhou, Mounib Bahri, Bowen Liu, Thomas Fellowes, Rob Clowes, Hongjun Niu, Nigel D. Browning, Andrew I. Cooper

**Affiliations:** a Leverhulme Research Centre for Functional Materials Design, University of Liverpool Liverpool L7 3NY UK aicooper@liverpool.ac.uk; b Department of Chemistry and Materials Innovation Factory, University of Liverpool Liverpool L69 7ZD UK; c Albert Crewe Centre for Electron Microscopy, University of Liverpool Liverpool L69 3GL UK

## Abstract

Recently, we reported the reconstruction of two-dimensional (2D) to three-dimensional (3D) covalent organic frameworks (COFs) *via* base-catalyzed boronate ester to spiroborate linkage conversion. In that work, we tentatively attributed the interlayer close-packing in the 2D BPDA-COF as the main cause for the long reaction time—40 days—required to complete the structure reconstruction in *N*,*N*-diethylformamide (DEF). Here, we address this hypothesis by designing methyl-substituted 4,4′-biphenyldiboronic acid (BPDA) with large molecular twist to weaken the packing between boronate esters. Experiments show that the spiroborate COF formation is accelerated by increased molecular twist in three linear diboronic acids linkers, with the pure 3D spiroborate phase obtained in 3 days *via* reaction of Co(ii) 2,3,9,10,16,17,23,24-octahydroxyphthalocyaninato ((OH)_8_PcCo) in *N*,*N*-diethylformamide (DEF). Mechanistic studies reveal that methyl-substituted linear diboronic acids are more liable to protodeboronation, which also contributes to the accelerated spiroborate structure formation.

## Abstract 2

Accelerated formation of spiroborate COF in *N*,*N*-diethylformamide by reacting (OH)_8_PcCo with methyl substituted 4,4′-biphenyldiboronic acid.

## Graphical abstract

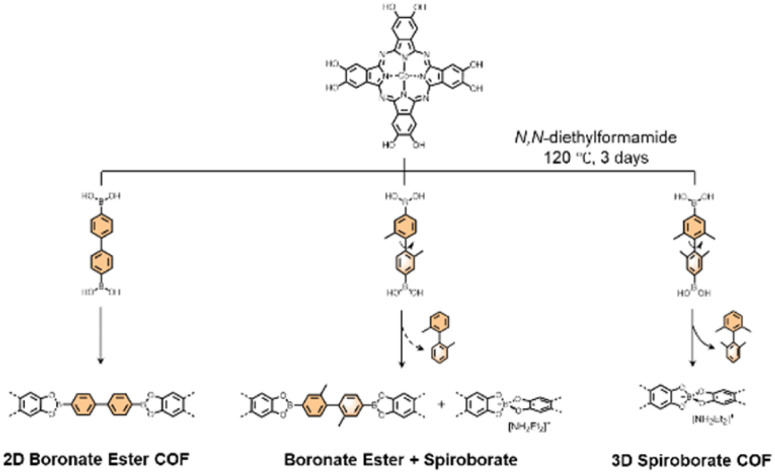

## Introduction

Covalent organic frameworks (COFs) have been explored for applications such as gas adsorption, catalysis, and energy storage.^1^ Boron, with the electronic configuration [He]2s^2^2p^1^, generally undergoes sp^2^ or sp^3^ hybridization to adopt trigonal planar or tetrahedral geometries, respectively, though rare examples of sp hybridization exist.^2,3^ In the field of COFs, the sp^2^ and sp^3^ hybridized boron are typically represented by neutral boronate ester^4^ and anionic tetraoxyborate^5,6^ or spiroborate linkage.^7,8^ According to our recent study, a 2D boronate ester COF—BPDA-COF—can be reconstructed to a 3D spiroborate COF—SPB-COF-DEA—under basic environments.^9,10^ When employing neat *N*,*N*-diethylformamide (DEF) as the solvent and reacting at 120 °C, 40 days are required to complete this structural transformation. Mechanistic studies have suggested a boronate ester to spiroborate linkage conversion *via* base-catalyzed boronate ester protodeboronation.^10^ According to the literature, the prehydrolysis of boronate ester to boronic acid is the rate determining step for this reaction.^11^ In prior reports, Dichtel *et al.* have discovered that the more closely-packed 2D boronate ester COFs exhibit improved hydrolytic stability because of the increased energy barriers to interlayer exfoliation and monomer release.^12^ Based on our mechanistic studies and the results of Dichtel *et al.*, we have tentatively assumed that it is the interlayer close-packing in 2D BPDA-COF that protected the boronate esters from hydrolysis, which then required 40 days to transform the 2D boronate ester COF to a pure 3D spiroborate COF in DEF. Here, we test this hypothesis by weakening the interlayer packing of 2D boronate ester COFs and explore its influence toward spiroborate COFs formation.

It is known that employing monomers of good planarity can enhance interlayer π–π stacking and facilitate the crystallization of 2D layered COFs.^12,13^ For example, 2D honeycomb-type COF with planar triazine core has showed much improved crystallinity and shortened interlayer d-spacing (3.49 Å) as compared to the structure analogue with a non-planar triphenylamine core (4.0 Å).^14^ Conversely, introducing twisted precursors usually results in diminished interlayer interactions that prevents effective packing between layers, as exemplified when highly twisted hexaazatrinaphthylene nodes (twisted-HATNA)^15^ or chiral 1,1′-bi-2-naphthol (BINOL) linker^16^ is employed for 2D-layered COFs synthesis. In either scenario, molecular twists are sterically enforced by rigid, bulky substitutions. According to these reports, we assume that by judicious precursor molecular conformation design, like, internal molecular twist introduction *via* substitutions, we can weaken or disrupt the interlayer packing of 2D BPDA-COF.

Specifically, BPDA-COF has been synthesized from the condensation between Co(ii) 2,3,9,10,16,17,23,24-octahydroxyphthalocyaninato ((OH)_8_PcCo) and 4,4′-biphenyldiboronic acid (BPDA).^10^ In BPDA-COF, although the square Co(ii) phthalocyanine (CoPc) ring with large aromatic domain is the major contributing part to the interlayer tight packing, structural modification of CoPc unit is synthetically challenging. We therefore propose to modulate the interlayer packing of BPDA-COF*via* twist introduction to the linear diboronic acid—BPDA.

**Fig. 1:**
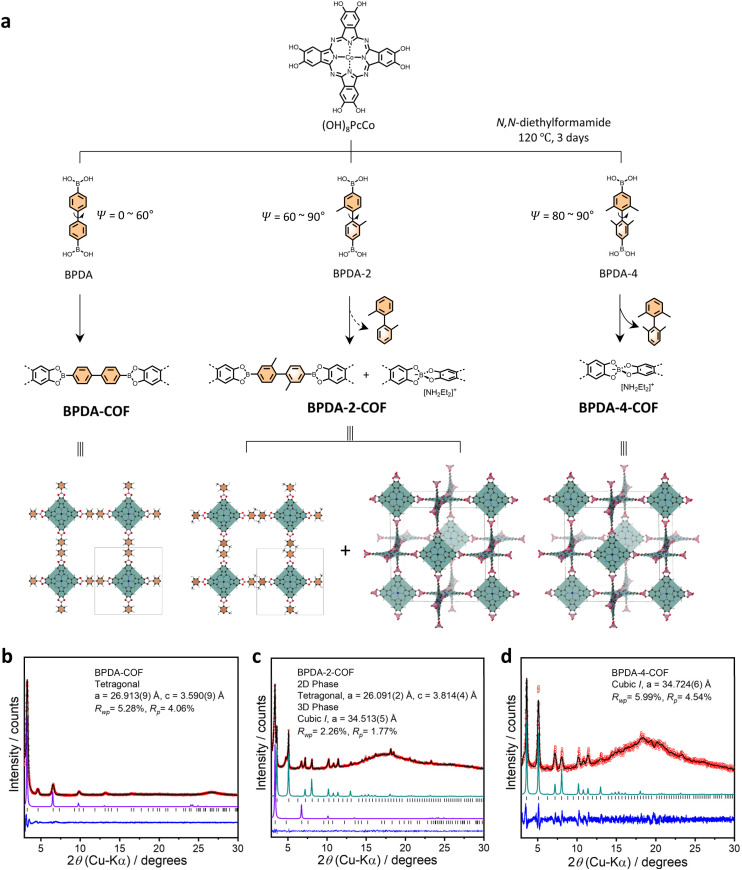
(a) Scheme for the synthesis of BPDA-COF, BPDA-2-COF and BPDA-4-COF with graph representations of their corresponding crystal models. The pink tetrahedra represents the spiroborate linkage and gray lines indicate the unit cell. Experimental PXRD pattern (red), profile calculated from Pawley fitting (black) showing the residual (blue), compared with the pattern simulated from the structural model (purple and green) for as-synthesized (b) BPDA-COF, (c) BPDA-2-COF and (d) BPDA-4-COF. Reflection positions are shown by tick marks.

By introducing two or four methyl substitutions at the 2,2′ or 2,2′,6,6′ positions on 4,4′-biphenyldiboronic acid (BPDA), we obtained two new linear diboronic acids, namely BPDA-2 and BPDA-4 (Fig. 1a). A survey of the known single crystal structures in the Cambridge Structural Database (CSD) revealed that the dihedral angle of biphenyl unit is 0–60° in linker BPDA, 60–90° in BPDA-2 and 80–90° in BPDA-4, suggesting increased molecular twist in order in the three linkers (Fig. S9†). Condensations of (OH)_8_PcCo with linker BPDA, BPDA-2 and BPDA-4 afforded BPDA-COF, BPDA-2-COF and BPDA-4-COF of 2D boronate ester structure, mixed 2D boronate ester and 3D spiroborate, and pure 3D spiroborate structure, respectively (Fig. 1). The corresponding COF structures was confirmed by a set of characterizations including powder X-ray diffraction (PXRD), Fourier-transform infrared (FTIR) spectroscopy and solid-state nuclear magnetic resonance (NMR). Mechanistic studies indicated that methyl-substituted BPDA-2 and BPDA-4 are more liable to protodeboronation, as compared to BPDA, which also contributes to the accelerated spiroborate COF formation.

## Results and discussion

### Synthesis and characterization of COFs

Linker BPDA-2 and BPDA-4 were synthesized from their corresponding amino precursors *via* a three-step procedure that involving Sandmeyer reaction to convert amino group to bromine, followed by Miyaura borylation and a subsequent boronate ester hydrolysis to give boronic acids. The amino precursor of BPDA-4 was synthesized from the reduction of 3, 5-dimethylnitrobenzene to obtain 1,2-bis(3,5-dimethylphenyl)hydrazine and a follow-up acid catalysed benzidine rearrangement (ESI†).^17^ Solvothermal condensation of (OH)_8_PcCo with linker BPDA, BPDA-2 and BPDA-4 in DEF at 120 °C for 3 days yielded BPDA-COF, BPDA-2-COF and BPDA-4-COF, respectively (Fig. 1a).

Experimental PXRD characterizations of the three COFs exhibited multiple well-defined diffraction peaks, suggesting high periodicity in two/three dimension (Fig. 1b–d). The experimental PXRD pattern of BPDA-COF was consistent with the reported 2D boronate ester crystal model of **sql** topology in AA-stacking mode, showing diffraction peaks at 3.26, 4.67, 6.60, 9.88, 13.15 and 16.52°, which were assigned to (100), (110), (200), (300), (400) and (430) planes. Pawley refinement confirmed a tetragonal lattice with unit cell parameters (*a* = 26.913(9) Å, *c* = 3.590(9) Å) (Fig. 1b).^9,10^ PXRD of BPDA-4-COF closely resembled our previously reported 3D spiroborate COF of non-interpenetrated **nbo** topology, indicating the formation of a pure spiroborate phase in BPDA-4-COF.^8,10^BPDA-4-COF showed diffraction peaks at 3.62, 5.13, 7.23, 8.08, 10.25, 10.85, 11.47, 13.09, 14.73, 18.16, 20.67 and 23.29°, which were indexed as (110), (200), (220), (310), (400), (330), (420), (510), (530), (550), (811) and (910) planes, respectively. Pawley refinement yielded a cubic *I* centered lattice with unit cell parameters of *a* = 34.724(6) Å (Fig. 1d). By contrast, the PXRD of BPDA-2-COF resembled the combined patterns of BPDA-COF and BPDA-4-COF, indicating a mixed phase of 2D boronate ester and 3D spiroborate structures (Fig. 1c). Specifically, in BPDA-2-COF, diffractions at 3.36, 4.75 and 6.76° can be assigned to the (100), (110) and (200) planes of the corresponding 2D boronate ester crystal model of **sql** topology in AA-stacking mode. Pawley refinement confirmed a tetragonal lattice with unit cell parameters of *a* = 26.091(2) Å, *c* = 3.814(4) Å. Diffraction peaks at 3.60, 5.10, 7.22, 8.08, 10.22, 10.85, 11.42, 13.04, 14.49, 14.93, 16.19, 18.15, 18.50, 20.55 and 23.30° were indexed as (110), (200), (220), (310), (400), (330), (420), (510), (440), (530), (620), (710), (640), (800) and (910) planes of the 3D spiroborate structure, respectively. Pawley refinement yielded a cubic *I* centered lattice with unit cell parameters of *a* = 34.513(5) Å. Comparing this result with our prior study, while the formation of a pure 3D spiroborate COF from linker BPDA in DEF takes 40 days,^10^ here, the spiroborate COF formation was accelerated by reacting (OH)_8_PcCo with methyl-substituted BPDA-2 and BPDA-4. Typically, when BPDA-4 was employed as the linker, a pure spiroborate phase can be isolated after 3 days.

**Fig. 2:**
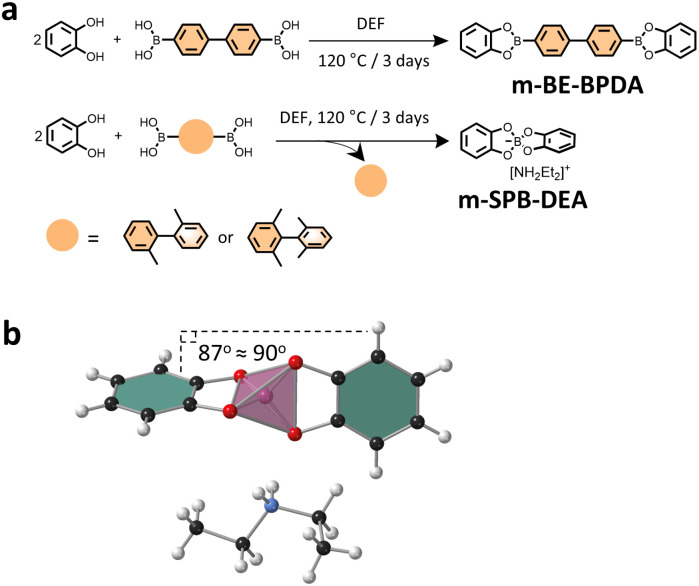
(a) Scheme for the synthesis of the model compounds. (b) Single crystal structure of m-SPB-DEA. The pink tetrahedron represents spiroborate linkage.

To gain insight into the type of linkage that was formed in the three COFs, model reactions were conducted by reacting catechol with the three linkers under COF synthesis condition. Notably, we observed that while the reaction between catechol and BPDA in DEF afforded pink-white coloured crystal precipitates, the reaction of catechol with BPDA-2 or BPDA-4 resulted in a homogeneous solution in brown colour. Solution ^1^H NMR analysis of the crystal precipitates confirmed a boronate ester product—m-BE-BPDA—in 56.4% yield, consistent with the boronate ester structure in BPDA-COF (Fig. 2a). Characterization of the isolated powder products from the reaction of catechol with BPDA-2 or BPDA-4 by solution ^1^H, ^13^C and ^11^B NMR identified a spiroborate structure—m-SPB-DEA, with the spiroborate linkage and the [NH_2_Et_2_]^+^ counter cations been validated by the single crystal structure of m-SPB-DEA (Fig. 2a and b). LC-MS analysis of the reaction mixture of catechol with BPDA-2 and BPDA-4 observed 2,2′-dimethyl-1,1′-biphenyl and 2,2′,6,6′-tetramethylbiphenyl as the reaction by-products, respectively (Fig. S8†). The results from model compound study were broadly the same as the result of COFs. While the reaction between catechol and linker BPDA-2 afforded solely m-SPB-DEA with spiroborate structure, we suppose the interlayer packing of the formed boronate esters protected the structure integrity of the boronate ester phase in BPDA-2-COF.

The chemical structure of the three COFs was further characterized by FTIR, solid-state carbon-13 cross-polarization/magic angle spinning NMR (^13^C CP/MAS NMR) and boron-11 magic angle spinning NMR (^11^B MAS NMR) spectroscopy. Like the three linkers, FTIR spectra of m-BE-BPDA and BPDA-COF showed a strong absorption band between 1329–1331 cm^−1^, corresponding to the stretching vibrations of the B–O of the trigonal [-BO_2_]. While FTIR spectra of BPDA-2-COF and BPDA-4-COF were consistent with the spectrum of m-SPB-DEA, showing characteristic B–O stretching vibrations of [BO_4_]^−^ tetrahedral at ∼1045 cm^−1^ (Fig. S11 and S12†).^10^ FTIR results corroborated the boronate ester structure of BPDA-COF and the formation of spiroborate linkage in BPDA-2-COF and BPDA-4-COF. In the solid-state ^13^C CP/MAS NMR spectra of the three COFs, signals between 154.9 and 95.8 ppm corresponding to phthalocyanine carbons were identified. The carbon signal at 16.6 ppm in BPDA-2-COF corresponded to methyl carbon in linker BPDA-2, while the carbon signals at 42.2 and 12.3 ppm in BPDA-2-COF, at 43.6 and 13.7 ppm in BPDA-4-COF were assigned to the secondary and primary carbons in the ethyl group of [NH_2_Et_2_]^+^ counter cation (Fig. S13–15†).^8,10^ Solid-state ^11^B MAS NMR spectra of BPDA-COF and BPDA-4-COF showed a single signal at 19.96 and 11.41 ppm, consistent with the same 2D boronate ester and 3D spiroborate COF reported before.^10^BPDA-2-COF exhibited a single signal at 16.16 ppm in ^11^B NMR spectrum, suggesting a mixed boronate ester and spiroborate linkage (Fig. S16†). The thermal stability of the three COFs was investigated by thermogravimetric analysis (TGA) (Fig. S17†). TGA curve of BPDA-COF showed around 5% weight loss at 500 °C and another 35% weight loss when heated up to 950 °C under N_2_ atmosphere. TGA analysis of BPDA-4-COF revealed about 10% weight loss at 200 °C, which corresponds to the loss of guest molecules (*e.g.*, H_2_O, DEF).^18,19^ The 20% weight loss between 200–450 °C was possibly from the loss of [NH_2_Et_2_]^+^ cation in the COF pores,^20,21^ and the 20% weight loss above 450 °C may arise from the decomposition of the COF.^22^ TGA curve of BPDA-2-COF was similar to BPDA-4-COF but with slightly higher thermal stability.

**Fig. 3:**
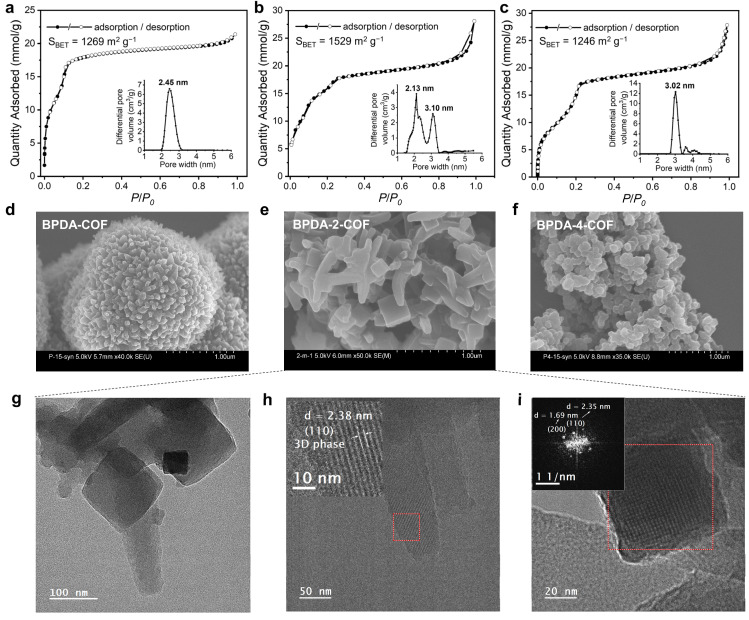
N_2_ sorption isotherms and the corresponding pore size distribution (PSD) profiles were calculated from DFT, and SEM images of (a and d) BPDA-COF, (b and e) BPDA-2-COF and (c and f) BPDA-4-COF were shown. (g–i) TEM images of BPDA-2-COF. The rod-shaped and cube-shaped morphologies were characterized respectively. Inset of (i) is the Fast Fourier Transform (FFT) of the area within red frame in (i).

The porosity of the three COFs was evaluated by nitrogen sorption measurements at 77 K (Fig. 3a–c). The Brunauer–Emmett–Teller (BET) surface areas were calculated to be 1269, 1529 and 1246 m^2^ g^−1^ for BPDA-COF, BPDA-2-COF and BPDA-4-COF. By fitting the density functional theory (DFT) model to the N_2_ isotherm, the derived pore size distribution (PSD) was found to be centred at 2.45 and 3.02 nm for BPDA-COF and BPDA-4-COF, supporting the 2D boronate ester structure of BPDA-COF and the 3D spiroborate structure of BPDA-4-COF.^10^ PSD of BPDA-2-COF revealed dual pores located at 2.13 and 3.10 nm, which corresponds to the 2D boronate ester and 3D spiroborate phase in BPDA-2-COF, respectively. The higher BET surface area of BPDA-2-COF than BPDA-4-COF was possibly due to the higher crystallinity of the sprioborate phase in BPDA-2-COF.

Scanning electron microscopy (SEM) images of the 2D BPDA-COF showed an overall flower-like morphology assembled from multiple rods (Fig. 3d). While the literature have shown that SEM images of the same spiroborate COF can have uniform cubic morphology,^8^ SEM image of BPDA-4-COF revealed small particles with particle size of ∼100 nm (Fig. 3f). SEM image of BPDA-2-COF showed a mixed morphology of rods and cubes (Fig. 3e). By comparing with the SEM image of the 2D BPDA-COF and the 3D spiroborate COF, we attributed the rod-like morphology to the 2D boronate ester phase and the cubes to the 3D spiroborate phase. However, transmission electron microscope (TEM) analysis of BPDA-2-COF revealed that both the cubes and the rods corresponds to the 3D spiroborate phase (Fig. 3g–i). Specifically, fast fourier transform (FFT) of the cubes clarified d-spacing of 2.35 and 1.69 nm, corresponding to (110) and (200) planes of the 3D spiroborate COF model. While the identified d-spacing of 2.38 nm of the rods can be assigned to the (110) plane of the 3D spiroborate phase. We did not observe the 2D phase from our TEM measurements. TEM images of BPDA-COF showed crystalline domains with identified d-spacing of 2.70 nm, corresponding to the (100) plane of the proposed 2D boronate ester structure model. TEM test for the crystalline structure of BPDA-4-COF was unsuccessful (Fig. S24–S26†).

### Mechanistic study

**Fig. 4:**
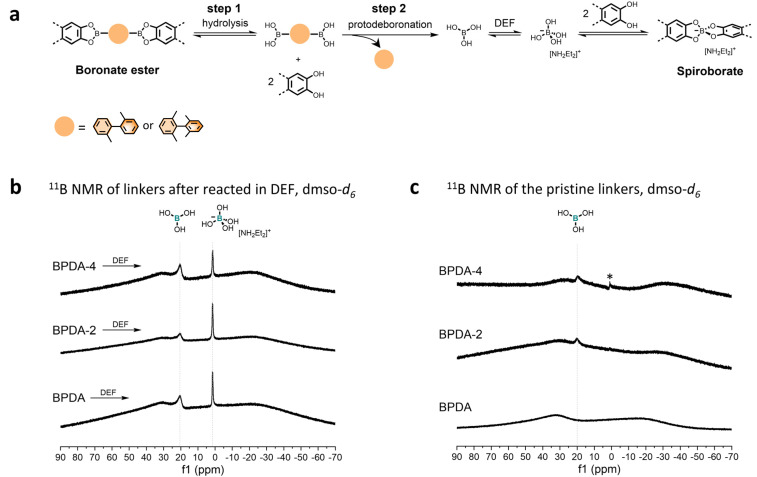
(a) Proposed scheme for spiroborate structure formation. (b) Solution ^11^B NMR of linker BPDA, BPDA-2 and BPDA-4 after reacted in DEF at 120 °C for 3 days. (c) Solution ^11^B NMR of pristine BPDA, BPDA-2 and BPDA-4. *The weak, sharp signal at ∼1.0 ppm in the ^11^B NMR spectra of linker BPDA-4 can be assigned to tetrahydroxyborate ion. Solution ^11^B NMR were conducted in quartz NMR tube.

Regarding the spiroborate structure formation in BPDA-2-COF and BPDA-4-COF, one potential route is *via* boronate ester to spiroborate linkage conversion.^10^ This route involves the prehydrolysis of the formed boronate esters to liberate the corresponding diol and boronic acid. Under basic environments, boronic acids undergo protodeboronation to release anionic [B(OH)_4_]^−^,^11,23^ which will react with diols to form spiroborate structures (Fig. 4a). In this study, we observed that the methyl-substituted linker BPDA-2 and BPDA-4 were capable to release anionic [B(OH)_4_]^−^ in DEF as the previously reported linker BPDA.^10^ Specifically, after subjecting BPDA-2 and BPDA-4 to DEF at 120 °C for 3 days, ^11^B NMR spectra of their reaction mixture showed one broad signal at ∼20.00 ppm and a sharp peak at 1.48 ppm, corresponding to the sp^2^ hybridized boron in boric acid (B(OH)_3_) and the sp^3^ hybridized boron in anionic [B(OH)_4_]^−^, respectively (Fig. 4b).^10^ LC-MS analysis of the reaction mixtures detected 2,2′-dimethyl-1,1′-biphenyl (from BPDA-2) and 2,2′,6,6′-tetramethyl-1,1′-biphenyl (from BPDA-4) as the reaction by-products, respectively, confirming protodeboronation occurred (Fig. S28†). This result obtained is in good agreement with the previous report and supported the feasibility of spiroborate structure formation in BPDA-2-COF and BPDA-4-COF*via* a linkage conversion way.

If we consider that methyl-substitutions onto linker BPDA only introduce steric effects to interfere with the effective packing between formed boronate esters and assuming the linkage conversion from boronate ester the only route to form spiroborate structure, then the above results support our initial assumption that the spiroborate COF formation can be accelerated by weakening or disrupting the packing of boronate esters. If this is the case, the accelerated spiroborate COF formation in BPDA-2-COF and BPDA-4-COF is due to the promoted monomer release—the hydrolysis step in Fig. 4a (step 1).

However, further characterizations indicate that methyl-substitutions onto the linker BPDA also contribute to the accelerated spiroborate COF formation by promoting the protodeboronation of linear diboronic acids (step 2 in Fig. 4a) – that is, the reactivity of the linker is affected. While we did not observe the signal of B(OH)_3_ in the solution ^11^B NMR spectrum of BPDA, ^11^B NMR spectra of linker BPDA-2 and BPDA-4 showed a weak signal at ∼20.00 ppm, suggesting protodeboronation occurred at room temperature in dmso-d_6_ (Fig. 4c). In addition, LC-MS test of the reaction mixture of linkers in neutral condition (1,4-dioxane : methanol = 2 : 1, v/v) revealed that the concentration of protodeboronation by-product from linker BPDA-2 (2,2′-dimethyl-1,1′-biphenyl) was higher than BPDA (biphenyl) (Fig. S31†), though both samples were prepared at a concentration of 1 mg. mL^−1^. These results together suggest that BPDA-2 and BPDA-4 are more liable to protodeboronation than BPDA. Such methyl substitution promoted protodeboronation of boronic acids is consistent with literature conclusions that electron-donating groups like methyl groups can increase protodeboronation liability of arylboronic acids.^24^

Based on these data, we propose that the accelerated spiroborate COF formation by reacting (OH)_8_PcCo with methyl-substituted BPDA-2 and BPDA-4 is due to a synergistic effect of the weakened or disrupted packing between the formed boronate esters that promoted the release of boronic acids and, the increased protodeboronation liability of boronic acids by methyl substitutions. Depending on the rate of boronate ester formation and the protodeboronation of dibornic acid linker, the protodeboronation of the linker may happen independently prior to boronate ester formation. Taking the synthesis of BPDA-2-COF as an example, on the one hand, as compared to 2D BPDA-COF, the employment of the twisted BPDA-2 weakened the interlayer packing of the formed boronate esters, which can facilitate boronate ester hydrolysis to release boronic acids (step 1 in Fig. 4a). On the other hand, methyl-substituted BPDA-2 is more prone to protodeboronation than BPDA to release B(OH)_3_ to form anionic [B(OH)_4_]^−^ for the formation of spiroborate structures in DEF (step 2 in Fig. 4a). These two factors together contribute to the accelerated spiroborate structure formation in BPDA-2-COF. This explains why a pure spiroborate phase can be achieved in 3 days when employing BPDA-4 as the linker, as BPDA-4 shows the largest molecular twist that can effectively prohibit the packing between boronate esters.

## Conclusions

In this work, the introduction of two or four methyl substitutions to 4,4′-biphenyldiboronic acid (BPDA) increases the precursor molecular twist, accelerating spiroborate COF formation in DEF. By reacting (OH)_8_PcCo with the three linkers BPDA, BPDA-2 and BPDA-4 with sequentially increased molecular twists, we realize accelerated spiroborate COF formation in order of the degree of twist. Mechanistic studies indicate that methyl substitution also contributes to the accelerated spiroborate COF formation in a way of promoting the protodeboronation liability of boronic acid. This work deepens our understanding toward boron chemistry in COF chemistry and can potentially inspire further research on exploring the protodeboronation mechanism of methyl-substituted aryl boronic acids.

## Author contributions

The manuscript was written through contributions of all authors. All authors have given approval to the final version of the manuscript.

## Conflicts of interest

There are no conflicts to declare.

## Supplementary Material

TA-013-D5TA02297E-s001

## Data Availability

The data that support the findings of this study are presented in the paper and the ESI.†
