# Supplementary material for: Influence of methyl substitution on linear diboronic acids: toward spiroborate covalent organic framework formation in N,N-diethylformamide
Source: J Mater Chem A Mater. 2025 May 21;13(25):19374–80. doi: 10.1039/d5ta02297e (PMC12108698; doi:10.1039/d5ta02297e)
Supplement: TA-013-D5TA02297E-s001 [file TA-013-D5TA02297E-s001.pdf]

Supplementary Information (SI) for Journal of Materials Chemistry A.
This journal is © The Royal Society of Chemistry 2025

Supporting Information for

Influence of Methyl Substitution on Linear Diboronic Acids: Toward Spiroborate Covalent Organic Framework Formation in *N,N*-Diethylformamide

Xue Wang,[†‡] Qiang Zhu,[†‡] Hang Qu,[‡] Xiang Zhou,[‡] Mounib Bahri,[§] Bowen Liu,[‡] Thomas Fellowes,[†‡] Rob Clowes,[‡] Hongjun Niu,[‡] Nigel D. Browning,[§] Andrew I. Cooper*[†‡]

[†] Leverhulme Research Centre for Functional Materials Design, University of Liverpool, Liverpool, L7 3NY, UK.

[‡] Department of Chemistry and Materials Innovation Factory, University of Liverpool, Liverpool, L69 7ZD, UK.

[§] Albert Crewe Centre for Electron Microscopy, University of Liverpool, Liverpool, L69 3GL, UK.

Table of Contents

1. Materials and Methods **3**

1.1 Solution nuclear magnetic resonance 3

1.2 Solid-state ^{13}C CP MAS and ^{11}B MAS nuclear magnetic resonance 3

1.3 High resolution mass spectrometry 3

1.4 Elemental Analysis 3

1.5 Liquid chromatography-mass spectrometry (LC-MS) Analysis 3

1.6 Inductively coupled plasma optical emission spectrometry 3

1.7 Powder X-ray diffraction 4

1.8 Single Crystal X-ray Diffraction 4

1.9 Fourier-transform infrared spectroscopy 4

1.10 Thermogravimetric analysis 4

1.11 Scanning electron microscopy 4

1.12 Transmission electron microscopy 4

1.13 Gas sorption analysis 4

2. Synthetic procedures **5**

2.1 Synthesis of diboronic acid linkers 5

2.2 Synthesis of COFs 9

2.3 Synthesis of model compounds 11

3. Conformations reported for (methyl substituted) biphenyl unit in the Cambridge Structural Database (CSD) **17**

4. Single Crystal Structures **18**

5. Fourier-transform infrared spectroscopy **20**

6. Solid state NMR spectra **22**

7. Thermogravimetric analysis **24**

8. Gas sorption isotherms **25**

9. Scanning electron microscopy **26**

10. Transmission electron microscopy **27**

11. Structural modelling **30**

12. Mechanistic study **35**

13. Solution NMR spectra of precursors **40**

14. Control study of $(OH)_8PcCo$ reacts with $B(OH)_3$ in DEF **48**

15. References **49**

1. Materials and Methods

$(OH)_8PcCo$ was synthesized according to reported procedure.[1, 2] Linker BPDA was obtained from TCI Europe. All reagents were obtained from Sigma-Aldrich, Manchester Organics, or TCI Europe. Anhydrous solvents were purchased from Sigma-Aldrich, Acros Organics or Fisher Scientific. All chemicals were used without further purification. All gases for sorption analysis were supplied by BOC at a purity of ≥99.9%. Reactions were carried out under nitrogen atmosphere using standard Schlenk techniques.

1.1 Solution nuclear magnetic resonance

NMR spectra were recorded on a Bruker Avance 400 NMR spectrometer, operated at frequencies of 400 MHz (^{1}H) and 100 MHz (^{13}C) and referenced against the residual ^{1}H or ^{13}C signal of the solvent. ^{11}B spectra were operating at a frequency of 128MHz using deuterium lock for reference. Solution ^{11}B NMR measurements were conducted in quartz NMR tubes.

1.2 Solid-state ^{13}C CP MAS and ^{11}B MAS nuclear magnetic resonance

Solid-state NMR experiments were performed on a Bruker Avance III HD spectrometer using the Durham University (UK) solid-state NMR service. Carbon-13 magic-angle spinning measurements were carried out at 100.63 MHz using a Bruker Avance III HD spectrometer and 4 mm (rotor o.d.) probe. Spectra were acquired at a spin rate of 10 kHz. Cross-polarisation (CP) spectra were recorded with TOSS spinning sideband suppression, 0.8 ms contact time and with a recycle delay of 1 s. Carbon spectra were measured relative to the chemical shift of neat tetramethylsilane. Experiments were carried out by setting the high-frequency signal from an external sample of adamantane to 38.5 ppm. 50 Hz of line broadening was added to improve the signal to noise. Boron-11 magic-angle spinning measurements were carried out at 128.39 MHz using a Bruker Avance III HD spectrometer and 4 mm (rotor o.d.) probe. Spectra were acquired at a spin rate of 20 kHz. All direct excitation ^{11}B spectra were acquired with a 1 µs 30-degree solid pulse which was determined from a 6 µs solution pulse determined on BF_3/OEt_2. The spectra were acquired with a recycle delay of 1 s determined on the sample. Boron spectral referencing is relative to BF_3/OEt_2. Since the probe used has a boron background, a spectrum of an empty rotor was collected, and this was subtracted from all spectra.

1.3 High resolution mass spectrometry

High resolution mass spectrometry (HR-MS) as performed on an Agilent Technologies 6530B accurate-mass QTOF mixed ESI/APCI mass spectrometer (capillary voltage 4000 V, fragmentor 225 V) in positive-ion detection mode.

1.4 Elemental Analysis

CHN analysis was performed on a Thermo EA1112 Flash CHNS-O Analyzer using standard microanalytical procedures.

1.5 Liquid chromatography-mass spectrometry (LC-MS) Analysis

Liquid chromatography-mass spectrometry (LC-MS) measurements were conducted from Waters. Reaction mixtures were characterized using MeOH : H_2O=80 : 20 with 0.1% formic acid addition as eluent, use C_{18} column and tested at 40 ºC. All samples were prepared in MeOH.

1.6 Inductively coupled plasma optical emission spectrometry

Inductively coupled plasma optical emission spectrometry (ICP-OES) measurements were conducted on an ICP-OES Agilent 5110. Samples were digested in concentrated nitric acid (67-69%, trace metal analysis grade) by microwave, and then diluted using distilled water.

1.7 Powder X-ray diffraction

Laboratory powder X-ray diffraction (PXRD) data patterns were collected in transmission mode on samples held on thin Mylar film in aluminium well plates on a Panalytical Empyrean diffractometer equipped with a high throughput screening (HTS) XYZ stage, X-ray focusing mirror, and PIXcel detector, using Cu-Kα radiation. For HT screening, PXRD patterns were measured over the 2θ range 3-30° over 10 minutes.

1.8 Single Crystal X-ray Diffraction

Single crystal X-ray data for model compounds were measured on a Rigaku MicroMax-007 HF rotating anode diffractometer (Mo-Kα radiation, λ = 0.71073 Å, Kappa 4-circle goniometer, Rigaku Saturn724+ detector) and data reduction was performed using CrysAlisPro. Structures were solved with SHELXT and refined by full-matrix least squares on $|F^2|$ by SHELXL,[3, 4] interfaced through the programme OLEX2.[5] All non-H atoms were refined anisotropically and all H-atoms were fixed in geometrically estimated positions and refined using the riding model. For full refinement details, see Table S1.

1.9 Fourier-transform infrared spectroscopy

FTIR spectra were recorded on a Bruker Tensor 27 at room temperature with an ATR method.

1.10 Thermogravimetric analysis

TGA analysis was carried out using a TA Q5000IR analyzer with an automated vertical overhead thermobalance. Samples were heated at a rate of 10 °C/min under a dry nitrogen gas flow.

1.11 Scanning electron microscopy

SEM images were recorded using a Hitachi S-4800 cold field emission scanning electron microscope (FE-SEM). Samples were prepared by drop coating the COFs anhydrous acetone solution (pre-sonicated for 15 min at room temperature) on to the cleaned silicon wafer, which were then coated with Chromium using an Emitech K550X automated sputter coater for 15 seconds. Imaging was conducted at a working voltage of 5.0 kV and a working distance of 5-9 mm using a combination of upper and lower secondary electron detectors.

1.12 Transmission electron microscopy

TEM images were obtained on a JEOL 2100FCs microscopy equipped with a DELTA Cs corrector operated at 200 kV. Since COF materials are electron beam sensitive, the electron beam damage to the specimen was minimized as much as possible (in this study, the beam density during the observations was less than 500 electrons/(nm2·s)). A Gatan 894 CCD camera was used for digital recording of the TEM images. A single TEM image with an exposure time of 2 seconds or a sequence of images (up to 20 frames) was recorded, with a 1 or 2 second exposure time for each. After drift compensation, some frames can be superimposed to increase the signal-to-noise (SN) ratio for display.

The as-synthesized COF powders were dispersed in anhydrous acetone by sonication and drop-cast on a holey carbon film on 200 mesh copper grids.

1.13 Gas sorption analysis

Surface areas were measured by nitrogen sorption at 77.3 K. Powder samples were degassed offline at room temperature, followed by degassing on the analysis port under vacuum at room temperature for 12 hours. Isotherms were measured using a Micromeritics 2420 (BPDA-COF) or 2020 (BPDA-2-COF and BPDA-4-COF) volumetric adsorption analyzer, with sorption data analyzed by MicroActive software. Surface areas were calculated in the relative pressure (P/P_0) range from 0.05 to 0.30 of the adsorption branch.

2. Synthetic procedures

2.1 Synthesis of diboronic acid linkers

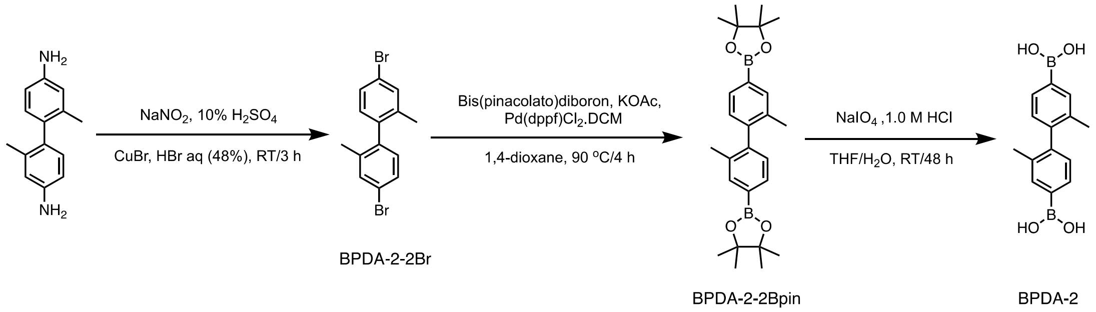

Scheme S1. Synthesis of linker **BPDA-2**.

The synthesis of **BPDA-2-2Br** followed a modified literature procedure.[6, 7]

Synthesis of BPDA-2-2Br: m-tolidine (1.06 g, 5.0 mmol, 1.0 equiv.) was dissolved in in 10% H_2SO_4 aq. (11.0 mL) at 10 °C and $NaNO_2$ (0.76, 11.0 mmol, 2.2 equiv.) in H_2O (6.2 mL) was added dropwise to the solution at 10 °C to form a diazonium salt. After stirring at 10 °C for 30 min, this diazonium salt solution was added dropwise to a cold solution of activated CuBr (7.17 g, 50.0 mmol, 10.0 equiv.) in 48% HBr aq. (69.0 mL) under ice bath. The reaction was slowly heated to 50 °C and stirred for 3 h (connected with an exhaust gas recirculation system with saturated sodium bicarbonate solution), after which it was cooled to room temperature. The solution was extracted with Et_2O (3 × 50 mL) (be careful!!! the mixing of the cooled reaction system with Et_2O is exothermic!!! For safety concern, recommend to operate the extraction step and the following washing in an open beaker or Erlenmeyer flask, rather than in the closed separating funnel). The Et_2O fractions were washed with 3.0 M HCl (until the HCl aq. became colourless) and H_2O (3 × 50 mL), then dried with anhydrous $MgSO_4$ and remove the solvent under reduced pressure. The product was purified by silica gel column chromatography (eluent = hexane) to give product as a transparent oil, which solidifies to white solids upon cooling down to room temperature. Yield 0.83 g, 49.0 %.

CuBr activation (the as-arrived CuBr from commercial supplier is of deep green colour)**:** CuBr was suspended in a very small amount of concentrated HCl (37%). The system was stirred at room temperature (rt.) for 30 min, which forms a dark green suspension. Then, large amount of distilled water was added into the suspension to precipitate the white CuBr powders, this suspension was stirred at rt. for 1.5 h and then filtrate. The filtrate cake was washed with distilled water and EtOH and then put at 80 °C vacuum oven overnight to obtain CuBr as pale-yellow powders.

^1H NMR (400 MHz, $CDCl_3$): δ 7.43 (d, J=4.0 Hz, 2H), 7.36 (dd, J_1=4.0 Hz, J_2=8.0 Hz, 2H), 6.94 (d, J=8.0 Hz, 2H), 2.03 (s, 6H) ppm. ^{13}C NMR (100 MHz, $CDCl_3$): δ 139.40, 138.25, 132.92, 130.87, 128.95, 121.45, 19.78 ppm. MS (ESI+) m/z calcd for $C_{14}H_{12}Br_2$ [M]+: 337.93. Found: 338.3421. Anal. Cald for $C_{14}H_{12}Br_2$: C: 49.45, H: 3.56. Found: C: 49.86, H: 3.51.

Synthesis of BPDA-2-2Bpin: BPDA-2-2Br (0.68 g, 2.0 mmol, 1.0 equiv.), bis(pinacolato) diboron (1.27 g, 5.0 mmol, 2.5 equiv.) and dry KOAc (0.59 g, 6.0 mmol, 3.0 equiv.) were suspended in anhydrous 1,4-dioxane (20 mL) and degassed for 30 min to remove air. Under nitrogen atmosphere, Pd(dppf)Cl_2·DCM (0.16 g, 0.2 mmol, 0.1 equiv.) was added and the mixture was stirred at 90 °C overnight. After that, the reaction mixture was cooled to rt., 10 mL H_2O was added to dilute the system and then using diethyl ether for extraction. Organic phase was combined, washed with water and then dried with anhydrous $MgSO_4$, with solvent removed by rotary evaporator. The resulting solid was purified via silica gel column chromatography using dichloromethane: petroleum ether (2:1, v/v) as the eluent. The solution obtained was evaporated to dryness to obtain white powders as the pure product. Yield 0.64 g, 74%.

^{1}H NMR (400 MHz, $CDCl_3$): δ 7.72 (s, 2H), 7.67 (d, *J*=8.0 Hz, 2H), 7.10 (d, *J*=8.0 Hz, 2H), 2.04 (s, 6H), 1.36 (s, 24H) ppm. ^{13}C NMR (100 MHz, $CDCl_3$): δ 144.75, 136.38, 135.09, 132.14, 128.67, 83.91, 25.03, 19.66 ppm. MS (ESI+) m/z calcd for $C_{26}H_{36}B_2O_4$ [M]+: 434.28. Found: 435.2882. Anal. Cald for $C_{26}H_{36}B_2O_4$: C: 71.92, H: 8.36. Found: C: 72.07, H: 8.50.

Synthesis of BPDA-2: BPDA-2-2Bpin (0.16 g, 0.37 mmol, 1.0 equiv.) was dissolved in THF/H_2O (v : v = 4:1, 15 mL) and sodium periodate ($NaIO_4$) (0.64 g, 3.0 mmol, 8.14 equiv.) was added. The suspension was stirred at rt. for 40 min, then 1M HCl (1.0 mL) was added, and the mixture was stirred at rt. for 48 h. The suspension was diluted with water (15 mL) and extracted with ethyl acetate (3 × 50 mL). The combined organic phases were washed with brine (3 × 10 mL) and H_2O (3 × 10 mL), dried over $MgSO_4$ and the solvent was removed under reduced pressure. The residue solids were washed with hexane (3 × 100 mL) and dried in vacuo to give product as white powders. Yield 0.09 g, 92%.

^{1}H NMR (400 MHz, dmso-d_6): δ 8.00 (s, 4H), 7.70 (s, 2H), 7.64 (d, *J*=8.0 Hz, 2H), 7.01 (d, *J*=8.0 Hz, 2H), 1.98 (s, 6H) ppm. ^{13}C NMR (100 MHz, dmso-d_6): δ 142.88, 135.77, 133.63, 131.48, 127.94, 19.49 ppm. MS (ESI+) m/z calcd for $C_{14}H_{16}B_2O_4$[M]+: 270.12. Found: 269.1162. Anal. Cald for $C_{14}H_{16}B_2O_4$: C: 62.30, H: 5.98. Found: C: 59.04, H: 4.88.

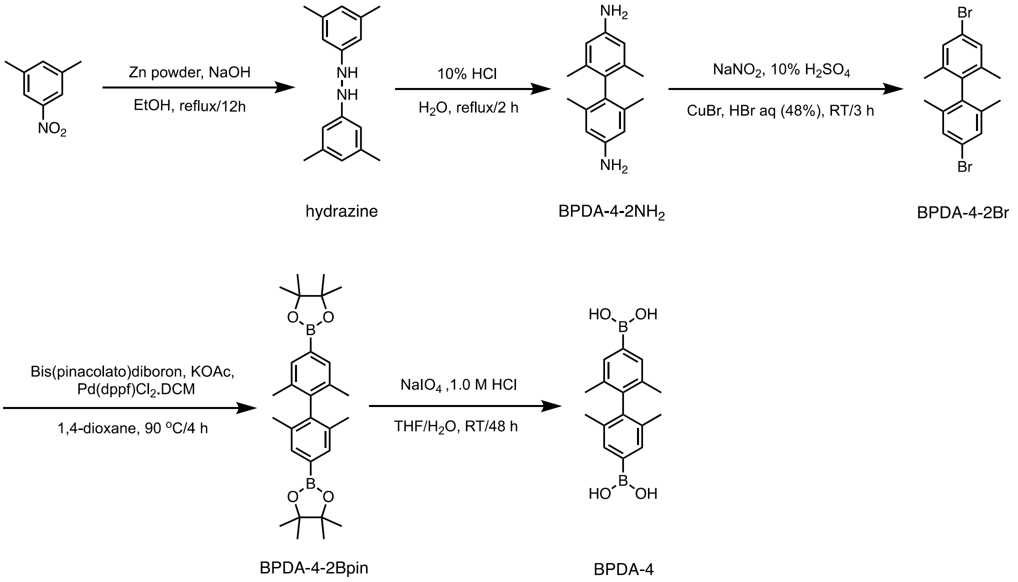

Scheme S2. Synthesis of linker **BPDA-4**.

The synthesis of hydrazine and **BPDA-4-2NH$_2$** followed a modified literature procedure.[6-8]

Synthesis of 1,2-bis(3,5-dimethylphenyl) hydrazine (hydrazine): A suspension of 3,5-dimethylnitrobenzene (10.0 g, 66.2 mmol, 1 equiv.), zinc powder (25.0 g, 384.0 mmol, 5.8 equiv.), and EtOH (40 mL) was heated to reflux over 30 min under N_2 atmosphere. A solution of sodium hydroxide (15.0 g, 377.0 mmol, 5.7 equiv.) in water (50 mL) was added dropwise to the zinc suspension to keep steady reflux, resulting in an orange solution with suspended zinc. Heating was continued at reflux overnight, while more zinc powder (10.0 g, 150.0 mmol, 2.3 equiv.) was added in three portions over the first 4 hours. After refluxed overnight, the hot suspension was then filtered over a bed of Celite into a solution of sodium bisulfite (1.0 g, 9.6 mmol, 0.15 equiv.) in 30% aqueous acetic acid (150 mL), and the filter cake was rinsed with hot EtOH. The slurry was cooled in an ice bath and filtered, yielding an orange solid, which was recrystallized from hot heptane (80 mL) to yield the desired pure product as large yellow crystals. Yield 6.0 g, 75%.

^{1}H NMR (400 MHz, $CDCl_3$): δ 6.50 (s, 6H), 5.46 (s, 2H), 2.25 (d, *J*= 4.0 Hz, 12H) ppm. ^{13}C NMR (100 MHz, $CDCl_3$): δ 149.39, 139.26, 121.87, 110.22, 21.51 ppm. MS (ESI+) m/z calcd for $C_{16}H_{20}N_2$ [M]+: 240.16. Found: 241.1703. Anal. Cald for $C_{16}H_{20}N_2$: C: 79.96, H: 8.39, N: 11.66. Found: C: 79.46, H: 8.43, N: 11.78.

Synthesis of 4,4’-diamino-2,2’,6,6’-tetramethylbiphenyl (BPDA-4-2NH$_2$): Hydrazine (0.8 g, 3.32 mmol) was added to a degassed (for 30 min) 10% HCl aq. (40 mL) and the reaction mixture was heated at reflux. The reaction was monitored by thin-layer chromatography (TLC) with ethyl acetate: hexane = 1: 2 as the eluent. After 2 h, TLC of the reaction mixture confirmed complete consumption of the starting materials. The reaction mixture was then cooled to room temperature and the pH was raised to >=10 with 1 M NaOH. The product was extracted using diethyl ether, washed with brine, dried over anhydrous $MgSO_4$ and concentrated in vacuo to afford brown-red coloured solid product. The crude product was purified by column chromatography on silica gel (ethyl acetate: hexane = 1: 2), to give pure product as pale pink-white powders. Yield 0.4 g, 50%.

^{1}H NMR (400 MHz, $CDCl_3$): δ 6.48 (s, 4H), 3.52 (s, 4H), 1.82 (s, 12H) ppm. ^{13}C NMR (100 MHz, $CDCl_3$): δ 144.72, 137.41, 130.82, 114.45, 20.13 ppm. MS (ESI+) m/z calcd for $C_{16}H_{20}N_2$ [M]+: 240.16. Found: 241.1704. Anal. Cald for $C_{16}H_{20}N_2$: C: 79.96, H: 8.39, N: 11.66. Found: C: 79.05, H: 8.42, N: 11.72.

Synthesis of BPDA-4-2Br: The synthesis of BPDA-4-2Br followed the same procedure for the synthesis of BPDA-2-2Br, gave white powder products with a yield of 40.0%.

^{1}H NMR (400 MHz, $CDCl_3$): δ 7.28 (s, 4H), 1.86 (s, 12H) ppm. ^{13}C NMR (100 MHz, $CDCl_3$): δ 137.88, 130.63, 121.00, 19.75 ppm. MS (ESI+) m/z calcd for $C_{16}H_{16}Br_2$[M]+: 365.96. Found: 366.9703. Anal. Cald for $C_{16}H_{16}Br_2$: C: 52.21, H: 4.38. Found: C: 52.53, H: 4.36.

Synthesis of BPDA-4-2BPin: The synthesis of BPDA-4-2BPin followed the same procedure for the synthesis of BPDA-2-2BPin, gave white powder products with a yield of 50.0%.

^{1}H NMR (400 MHz, $CDCl_3$): δ 7.57 (s, 4H), 1.88 (s, 12H), 1.37 (s, 24H) ppm. ^{13}C NMR (100 MHz, $CDCl_3$): δ 143.41, 134.79, 133.99, 83.86, 25.07, 19.68 ppm. MS (ESI+) m/z calcd for $C_{28}H_{40}B_2O_4$ [M]+: 462.31. Found: 463.3200. Anal. Cald for $C_{28}H_{40}B_2O_4$: C: 72.76, H: 8.72. Found: C: 72.37, H: 8.75.

Synthesis of BPDA-4: The synthesis of BPDA-4 followed the same procedure for the synthesis of BPDA-2, gave white powder products with a yield of 90.0%.

^{1}H NMR (400 MHz, dmso-d_6): δ 7.93 (s, 4H), 7.56 (s, 4H), 1.82 (s, 12H) ppm. ^{13}C NMR (100 MHz, dmso-d_6): δ 141.39, 133.48, 133.23, 19.53 ppm. MS (ESI+) m/z calcd for $C_{16}H_{20}B_2O_4$ [M]+: 298.15. Found: 301.1411. Anal. Cald for $C_{16}H_{20}B_2O_4$: C: 64.50, H: 6.77. Found: C: 64.68, H: 6.27.

2.2 Synthesis of COFs

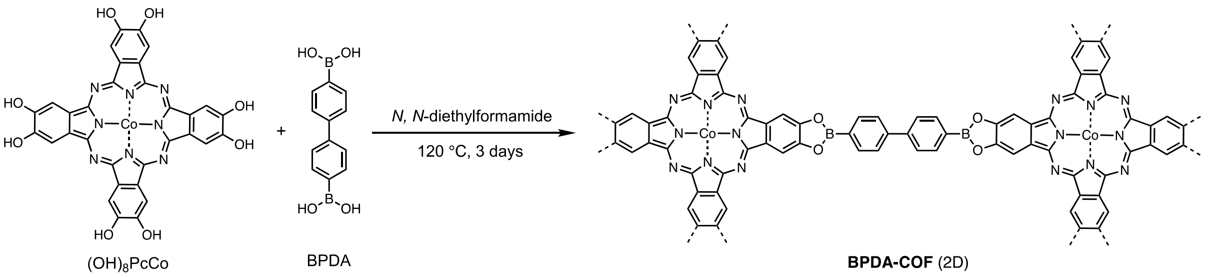

Scheme S3. Synthesis of **BPDA-COF**.

Synthesis of BPDA-COF: A 10 mL Pyrex tube was charged with $(OH)_8PcCo$ (10.5 mg, 0.015 mmol), BPDA (7.3 mg, 0.03 mmol) and *N,N*-diethylformamide (DEF) (1.0 mL). The mixture was sonicated at room temperature for 2 minutes, then flash frozen in a liquid N_2 bath and degassed through three freeze-pump-thaw cycles and sealed under vacuum using a Schlenk line and oil pump. Upon warming to rt., the tube was put into a 120 °C oven for 72 hours and then taken out of the oven, which yielded a black product. After cooling to rt., the product was washed with anhydrous acetone to give dark-coloured powder products. The obtained powder was immersed in anhydrous acetone, and the solvent was exchanged with fresh acetone three times with intervals of 24 hour. The wet sample was transferred to a Critical Point Drier (Quorum-E3100AG); the sample was then washed and exchanged with liquid CO_2 for 4 ~ 5 times with an interval of 1 ~ 2 hours until all acetone in the material was successfully exchanged. After the final exchange, the system was heated to reach the critical point and the supercritical CO_2 was then released slowly over 1 hour. After the chamber pressure returned to ambient, samples were then transferred into a gas adsorption test tube and degassed at room temperature for 12 h to afford BPDA-COF in 77% yield (12.0 mg) as black powders. This batch of COFs was used in all the experiments/measurements unless otherwise specified. For gas sorption and solid-state NMR measurements, several batches of BPDA-COF were combined together. Anal. Cald for $C_{60}H_{28}N_4B_4O_8Co$: C: 64.74; H: 2.33; N: 10.79. Found: C: 62.64; H: 5.28; N: 8.80. Theoretical Co content: 5.67 wt.%. ICP-OES analysis shows a Co content of 4.29 wt.%.

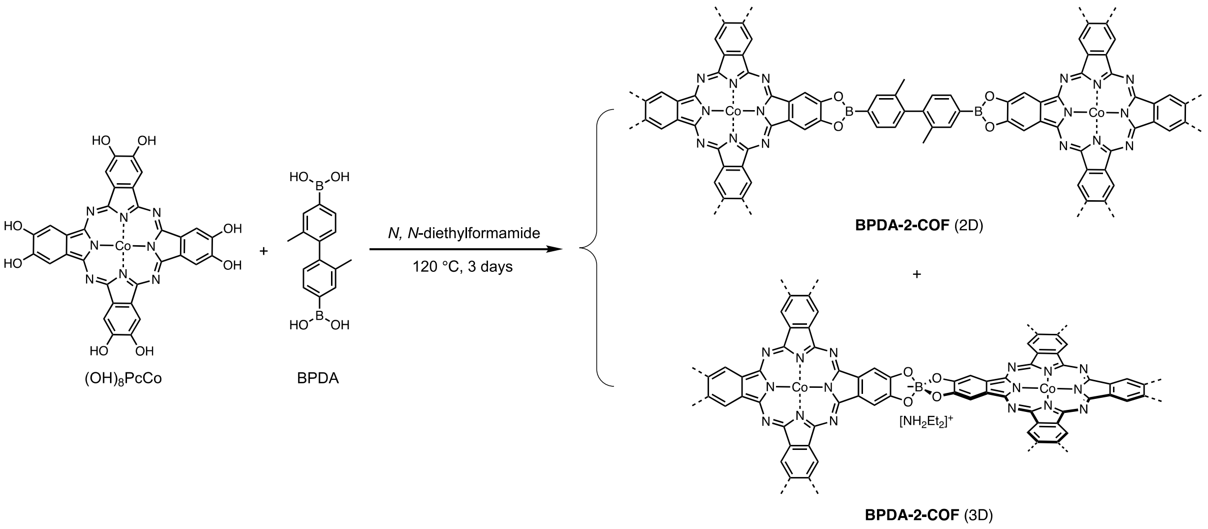

Scheme S4. Synthesis of **BPDA-2-COF**.

Synthesis of BPDA-2-COF: The synthesis of BPDA-2-COF followed the same procedure for the synthesis of BPDA-COF, but with BPDA-2 (8.1 mg, 0.03 mmol) as the linker. 14.0 mg BPDA-2-COF was obtained as black powders. For reference, theoretical 100% yield based-on boronate ester linkage is 16.4 mg and the theoretical 100% yield supposing a purely spiroborate structure is 12.9 mg. This batch of BPDA-2-COF was used in all the experiments/measurements unless otherwise specified. Anal.

Cald for $C_{60}H_{32}N_8O_8B_4Co$ (supposing 100% boronate ester linkage): C: 65.81; H: 2.95; N: 10.23. Anal. Cald for $C_{40}H_{32}N_{10}O_8B_2Co$ (supposing 100% spiroborate linkage): C: 55.78; H: 3.75; N: 16.26. Found: C: 54.25; H: 4.38; N: 10.54. Theoretical Co content: 5.38 wt.% (supposing 100 % boronate ester linkage) or 6.84 wt.% (supposing 100 % spiroborate linkage). ICP-OES analysis shows a Co content of 5.05 wt.%.

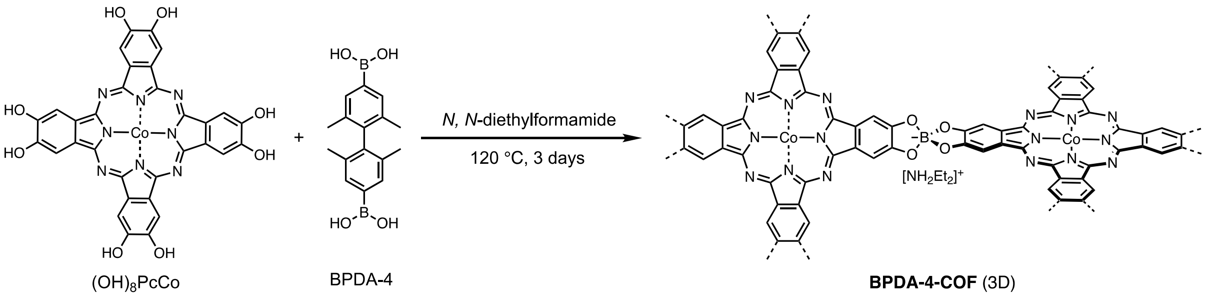

Scheme S5. Synthesis of **BPDA-4-COF**.

Synthesis of BPDA-4-COF: The synthesis of BPDA-4-COF followed the same procedure for the synthesis of BPDA-COF, but with BPDA-4 (9.0 mg, 0.03 mmol) as the linker. 9.0 mg BPDA-4-COF was obtained as black powders in 70% yield (calculated based on purely spiroborate linkage). This batch of BPDA-4-COF was used in all the experiments/measurements unless otherwise specified. Anal. Cald for $C_{40}H_{32}N_{10}O_8B_2Co$ (based-on 100 % spiroborate linkage): C: 55.78; H: 3.75; N: 16.26. Found: C: 50.12; H: 3.99; N: 15.31. Theoretical Co content: 6.84 wt.% (based-on 100 % spiroborate linkage). ICP-OES analysis shows a Co content of 5.44 wt.%.

2.3 Synthesis of model compounds

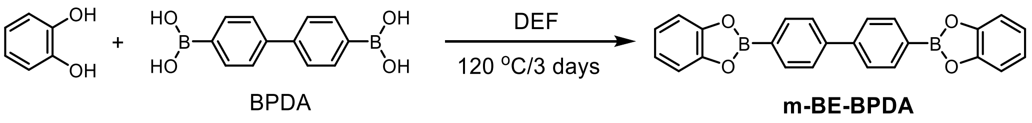

Scheme S6. Model reaction between catechol and linker BPDA in DEF.

Model reaction between catechol and linker BPDA: Under N_2 atmosphere, to a mixture of 4, 4'-biphenyldiboronic acid (BPDA) (0.121 g, 0.5 mmol, 1.0 equiv.) and 1, 2-dihydroxybenzene (= catechol) (0.115 g, 1.04 mmol, 2.08 equiv.) were added 12 mL of *N*,*N*-diethylformamide (DEF). The solution was stirred at 120 °C for 3 days with a Dean-Stark trap half filled with activated 3 Å molecular sieves. During this time a white solid began to precipitate from the transparent light-yellow solution. After the reaction mixture was cooled to room temperature, the white precipitate in the reaction system was collected through centrifugation and washed with hexane for three times, then dried in 80 °C vacuum oven for 6 hours to get 0.11 g m-BE-BPDA as white crystalline needles/powders in 56.4 % yield.

^{1}H NMR (400 MHz, $CDCl_3$): δ 8.20 (d, J = 8.0 Hz, 4H), 7.80 (d, J = 8.0 Hz, 4H), 7.34 (m, 4H), 7.15 (m, 4H) ppm. Due to the poor solubility of this compound in $CDCl_3$, no solution ^{13}C NMR spectrum was obtained. Anal. Cald for $C_{24}H_{16}B_2O_4$: C: 73.91, H: 4.14. Found: C: 72.31, H: 4.04.

*One thing needs to notify is, this model system had been repeated for several times under the same reaction condition to confirm the results. However, it turned out that from some batches of synthesis, this model reaction can yield a mixture of boronate ester and spiroborate structures, with their corresponding ratio varies from different batch of synthesis. Nevertheless, the boronate ester structure was always isolated as precipitates and the spiroborate structure in solution. We assume the formation of spiroborate structure from this model system in solution can be attributed to the weaker intermolecular packing (m-BE-BPDA) in solution compared with 2D COFs.

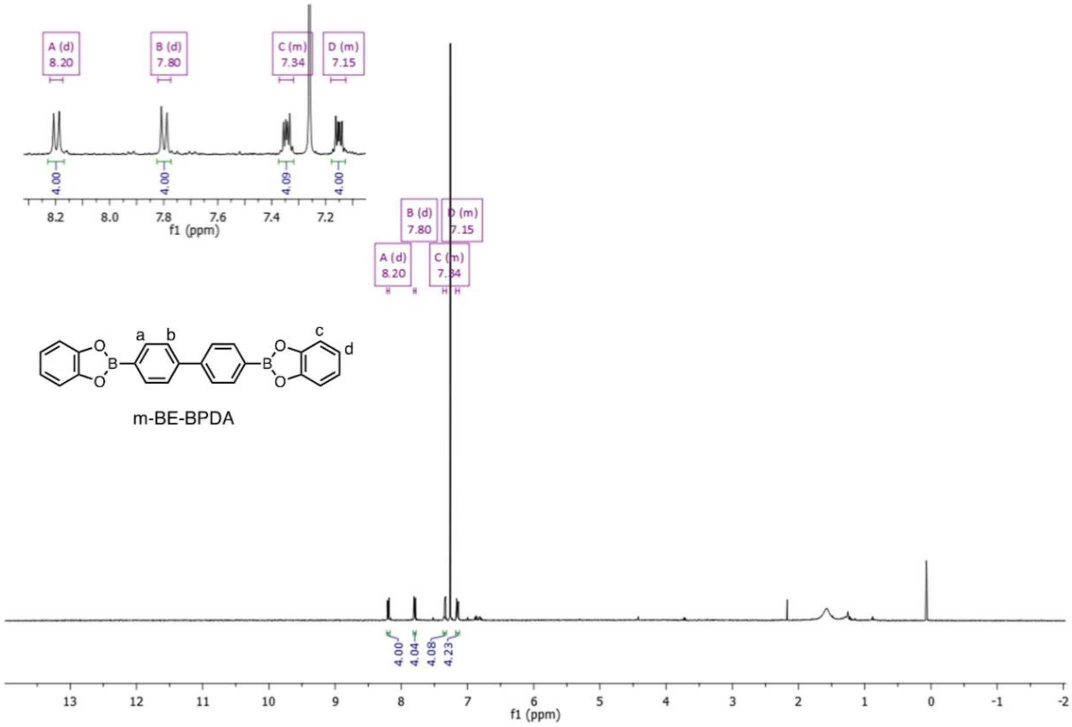

Figure S1. ^{1}H NMR spectrum of **m-BE-BPDA** in $CDCl_3$. Isolated from the reaction between catechol and linker BPDA.

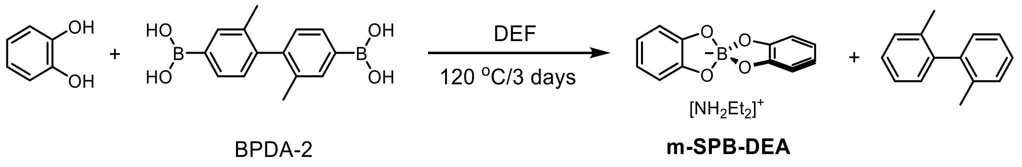

Scheme S7. Scheme for the model reaction between catechol and linker BPDA-2 in DEF.

Model reaction between catechol and linker BPDA-2: Under N_2 atmosphere, to a mixture of linker BPDA-2 (0.108 g, 0.4 mmol, 1.0 equiv.) and catechol (0.092 g, 0.832 mmol, 2.08 equiv.) were added 12 mL of DEF. The solution was stirred at 120 °C for 3 days with a Dean-Stark trap half filled with activated 3 Å molecular sieves. During this time, the reaction solution changed from a transparent light-yellow solution to an opaque dark brown solution (no precipitates formed). After the reaction mixture was cooled to rt., the solvent was removed using vacuum distillation. The brown oil residue was then dissolved in very small amount of dichloromethane and added dropwise to a stirred cold diethyl ether (300 mL) to precipitate the product. The solid product was collected by filtration and dried in a vacuum oven at 80 °C for 6 hours to give 0.078 g m-SPB-DEA as pale brown powders in 62.0% yield (yield calculated based on catechol). Single crystal of m-SPB-DEA suitable for X-ray diffraction were grown from its saturated ethyl acetate solution at rt. for a week.

^{1}H NMR (400 MHz, dmso-d_6): δ 8.13 (s, 2H), 6.47 (s, 8H), 2.92 (q, J = 8.0 Hz, 4H), 1.15 (t, J = 8.0 Hz, 6H) ppm. ^{13}C NMR (100 MHz, dmso-d_6): δ 151.55, 117.27, 107.58, 41.38, 11.05 ppm. ^{11}B NMR (128 MHz, dmso-d_6): δ = 14.20 ppm. MS (ESI+) m/z calcd for $[NH_2Et_2]^+$ [M]+: 74.15. Found: 74.0968. MS (ESI-) m/z calcd for $C_{12}H_8BO_4$ [M]−: 227.05. Found: 227.0526. Anal. Cald for $C_{16}H_{20}BNO_4$: C: 63.81, H: 6.69, N: 4.65. Found: C: 65.16, H: 4.96, N: 3.71.

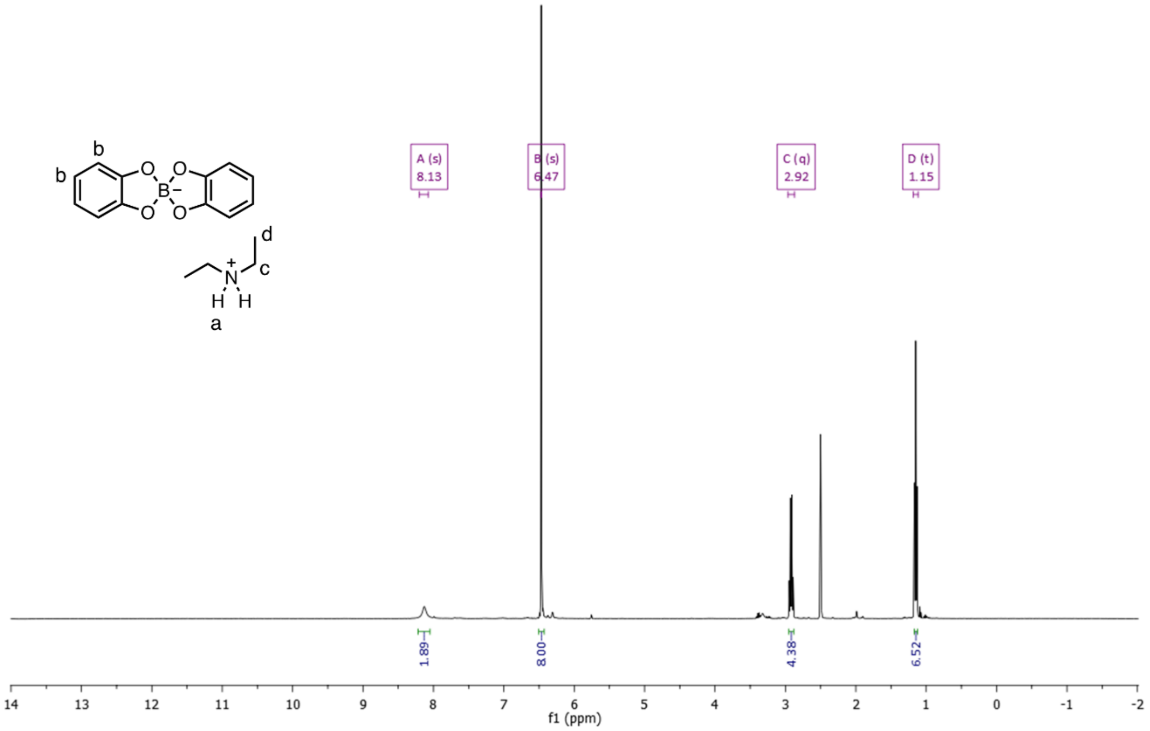

Figure S2. ^{1}H NMR spectrum of **m-SPB-DEA** in dmso-d_6. Isolated from the reaction between catechol and linker BPDA-2.

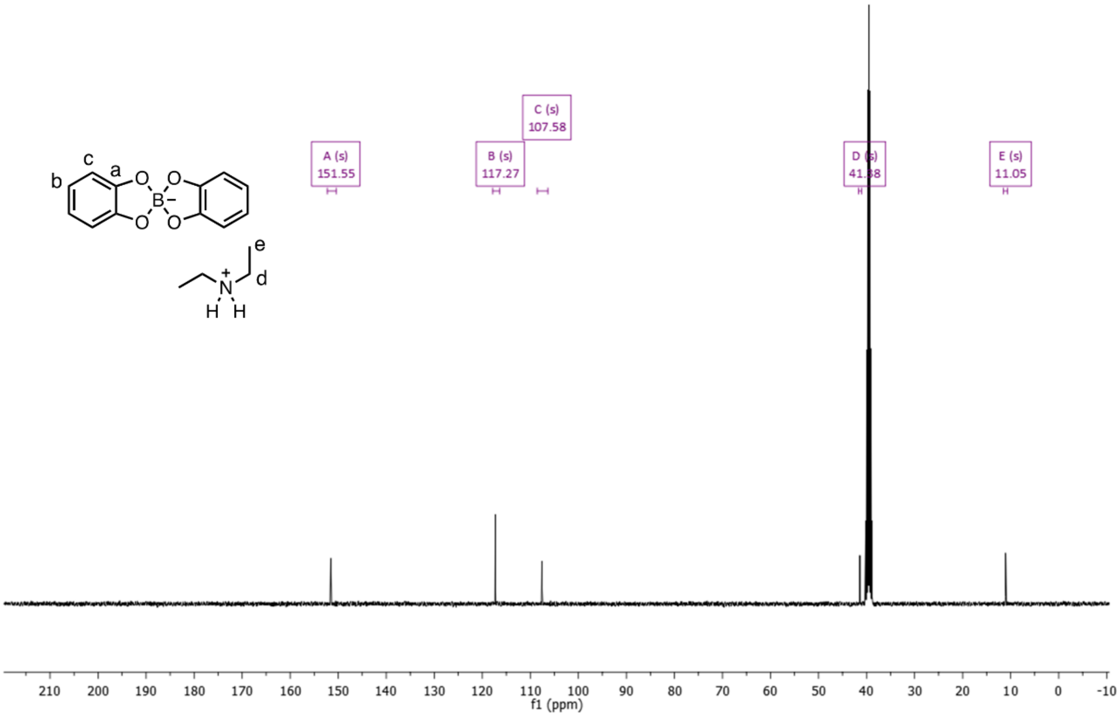

Figure S3. ^{13}C NMR spectrum of **m-SPB-DEA** in dmso-d_6. Isolated from the reaction between catechol and linker BPDA-2.

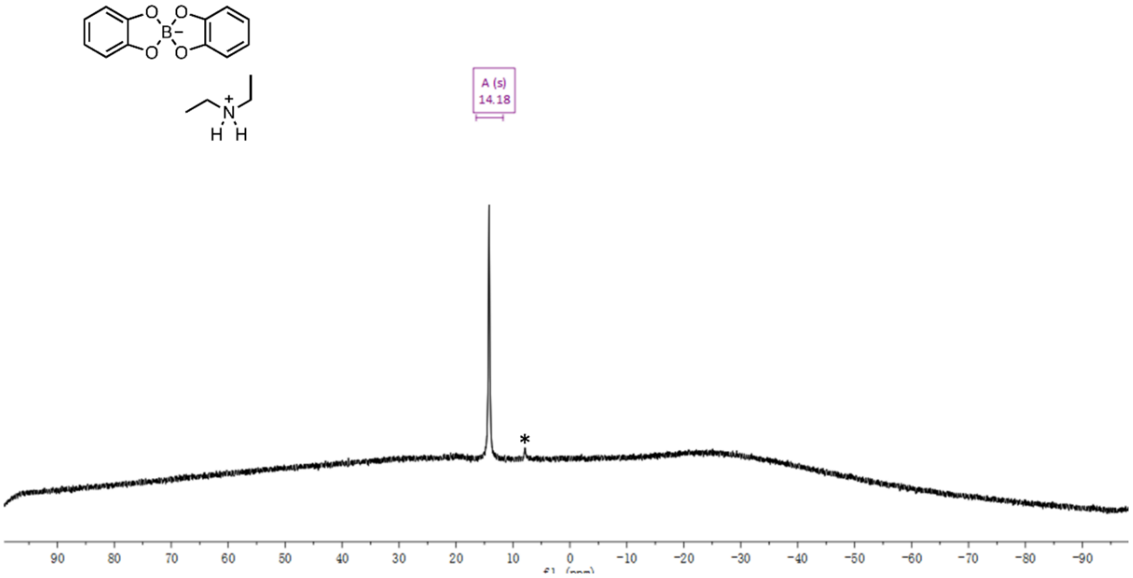

Figure S4. ^{11}B NMR spectrum of **m-SPB-DEA** in dmso-d_6. Isolated from the reaction between catechol and linker BPDA-2. *Unknown signal.

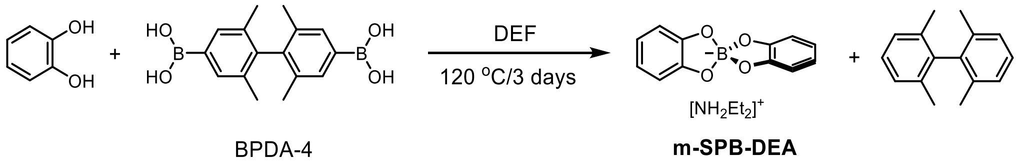

Scheme S8. Scheme for the model reaction between catechol and linker BPDA-4 in DEF.

Model reaction between catechol and linker BPDA-4: Under N_2 atmosphere, to a mixture of linker BPDA-4 (0.104 g, 0.35 mmol, 1.0 equiv.) and catechol (0.081 g, 0.728 mmol, 2.08 equiv.) were added 12 mL of DEF. The solution was stirred at 120 °C for 3 days with a Dean-Stark trap half filled with activated 3 Å molecular sieves. During this time, the reaction solution changed from a transparent light-yellow solution to an opaque dark brown solution (no precipitates formed). After the reaction mixture was cooled to rt., the solvent was removed using vacuum distillation. The brown oil residue was then dissolved in very small amount of dichloromethane and added dropwise to stirred cold diethyl ether (300 mL) to precipitate the product. The solid product was collected by filtration and dried in a vacuum oven at 80 °C for 6 hours to give 0.072 g m-SPB-DEA as pale brown powders in 65.7% yield (yield calculated based on catechol). Single crystals of m-SPB-DEA suitable for X-ray diffraction were grown from its saturated ethyl acetate solution at rt. for a week.

^{1}H NMR (400 MHz, dmso-d_6): δ 8.19 (s, 2H), 6.47 (s, 8H), 2.91 (q, J = 8.0 Hz, 4H), 1.15 (t, J = 8.0 Hz, 6H) ppm. ^{13}C NMR (100 MHz, dmso-d_6): δ 151.55, 117.27, 107.58, 41.38, 11.09 ppm. ^{11}B NMR (128 MHz, dmso-d_6): δ = 14.22 ppm. MS (ESI+) m/z calcd for $[NH_2Et_2]^+$ [M]+: 74.15. Found: 74.0967. MS (ESI-) m/z calcd for $C_{12}H_8BO_4$ [M]−: 227.05. Found: 227.0527. Anal. Cald for $C_{16}H_{20}BNO_4$: C: 63.81, H: 6.69, N: 4.65. Found: C: 65.16, H: 4.96, N: 3.71.

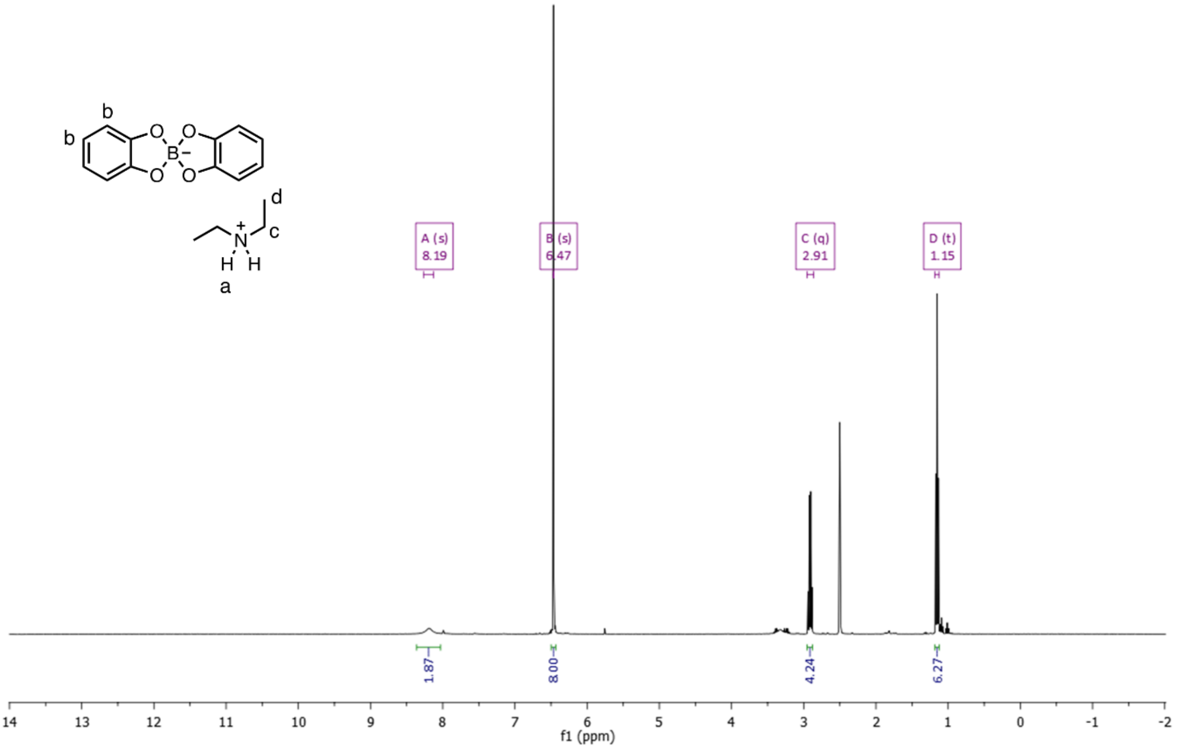

Figure S5. ^{1}H NMR spectrum of **m-SPB-DEA** in dmso-d_6. Isolated from the reaction between catechol and linker BPDA-4.

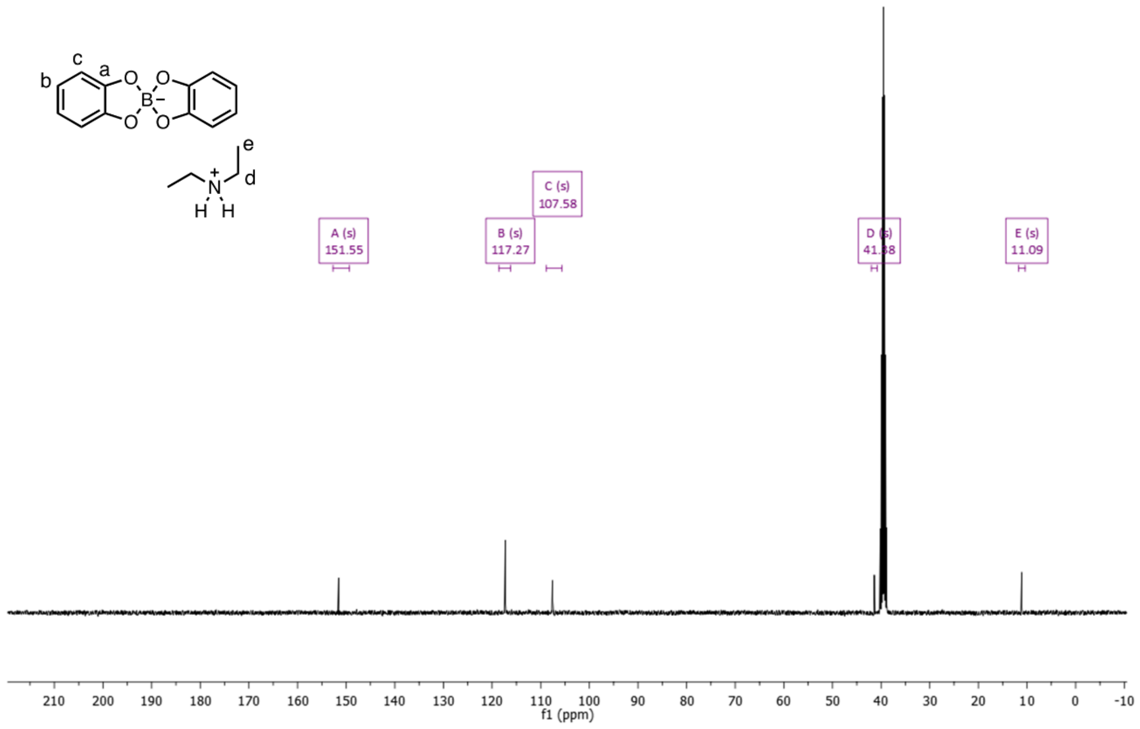

Figure S6. ^{13}C NMR spectrum of **m-SPB-DEA** in dmso-d_6. Isolated from the reaction between catechol and linker BPDA-4.

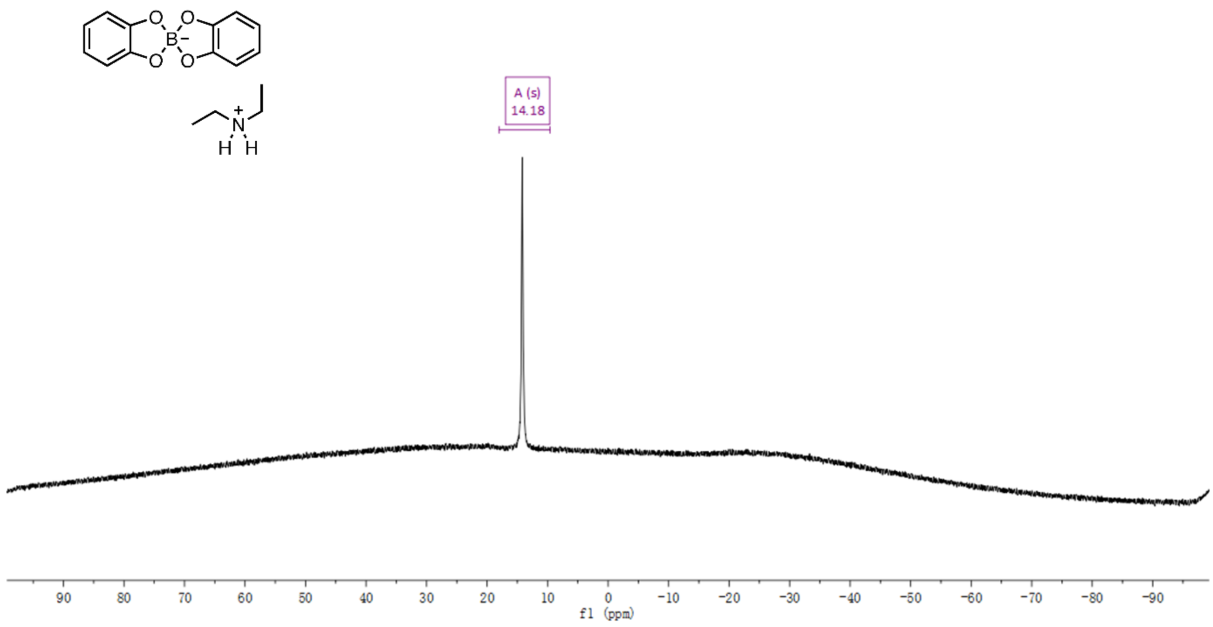

Figure S7. ^{11}B NMR spectrum of **m-SPB-DEA** in dmso-d_6. Isolated from the reaction between catechol and linker BPDA-4.

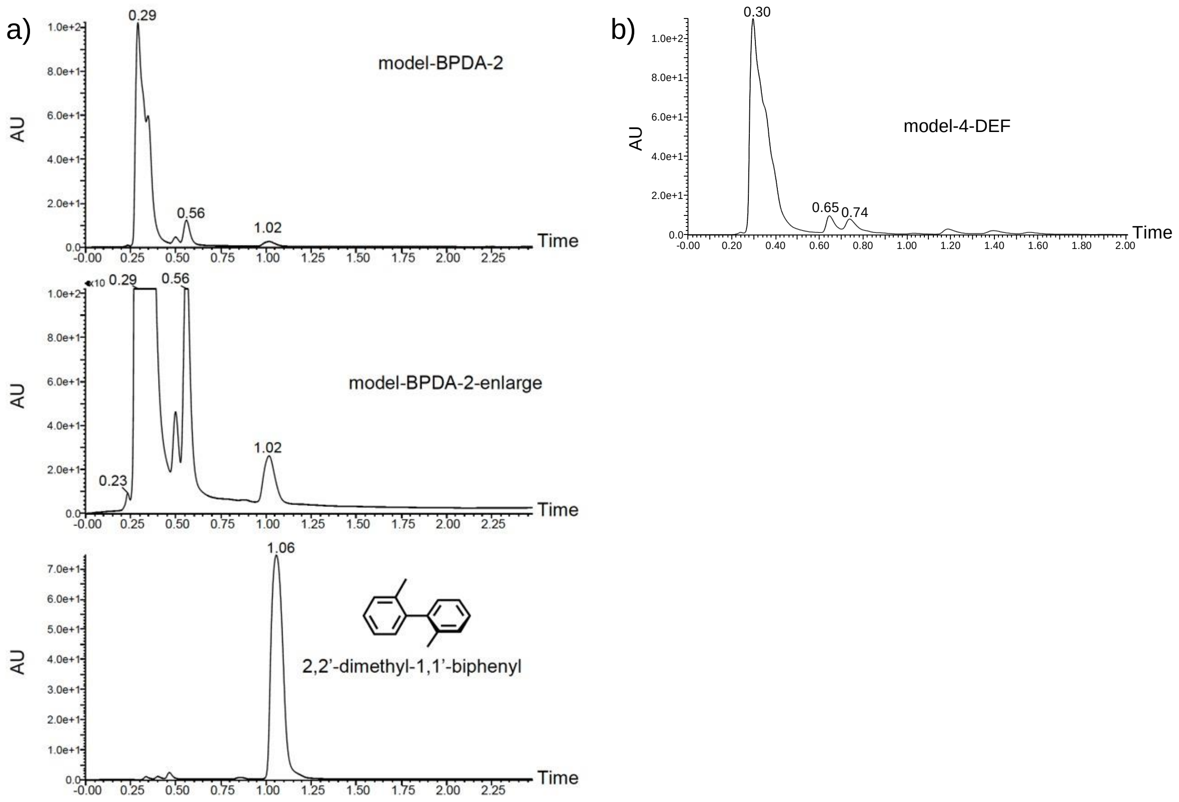

Figure S8. (a) LC of the reaction mixture of catechol reacts with linker **BPDA-2** in DEF and its comparison with 2,2'-dimethyl-1,1'-biphenyl reference. **(b)** LC of the reaction mixture of catechol reacts with linker **BPDA-4** in DEF. Comparing this figure with **Figure S28c** or **Figure S31c** can draw a conclusion that the signal at the time of 0.74 min corresponds to 2, 2',6, 6'-tetramethylbiphenyl, the protodeboronation by-product of linker **BPDA-4**.

3. Conformations reported for (methyl substituted) biphenyl unit in the Cambridge Structural Database (CSD)

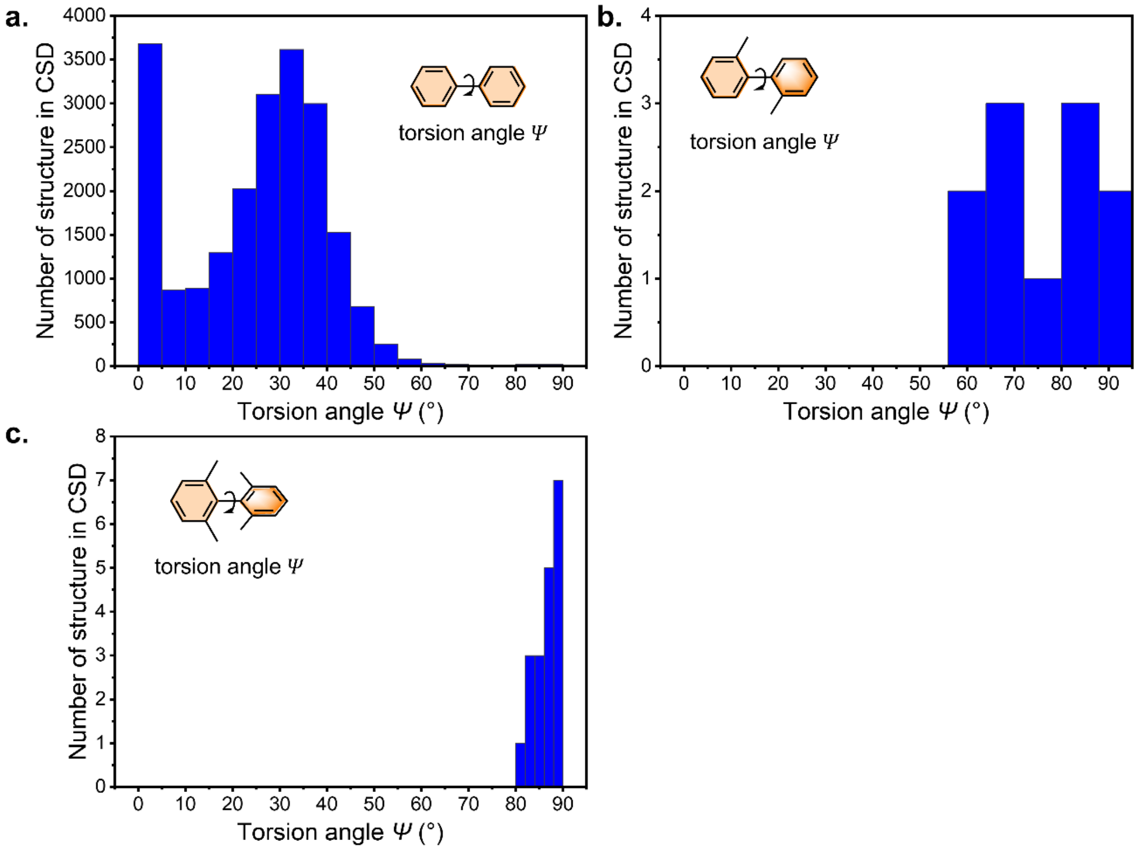

Figure S9. Distribution of the torsion angles expressed by **(a)** biphenyl unit. 4991 crystals hit. **(b)** 2, 2'-dimethyl-1, 1'-biphenyl unit. 11 crystals hit. **(c)** 2, 2', 6, 6'-tetramethyl-1, 1'-biphenyl unit. 14 crystals hit. Results are summarized from Cambridge Structure Database (CSD) in February 2025. The above conformer search result illustrated that via two or four methyl substitutions, the molecular twist of biphenyl unit can be increased in sequence in linker **BPDA**, **BPDA-2** and **BPDA-4**.

4. Single Crystal Structures

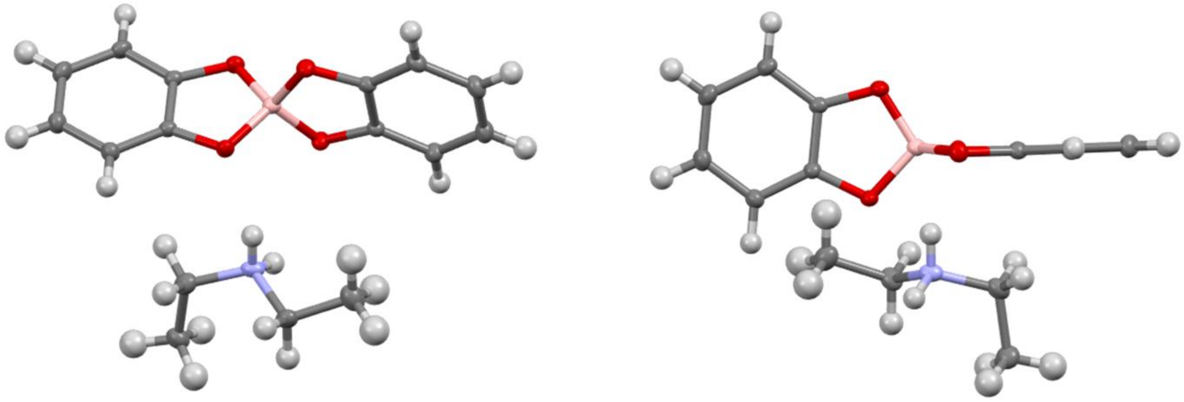

Figure S10. Displacement ellipsoid plots from the single crystal structure of **m-SPB-DEA**; two views are shown; ellipsoids are displayed at 50% probability level. C = grey; H = white; N = blue; O = red; B = pink. Single crystal structure of **m-SPB-DEA** from the reaction system of linker **BPDA-2** and **BPDA-4** are the same.

Table S1. Single crystal refinement details for **m-SPB-DEA**.

**Name**	**m-SPB-DEA**
Crystallization solvent	ethyl acetate
Wavelength/ Å	0.71073 (Mo-Kα)
Formula	$C_{12}H_8BO_4$, $C_4H_{12}N$
Weight	301.14
Crystal size/mm^3	0.238 × 0.225 × 0.173
Crystal system	monoclinic
Space group	$P2_1/n$
a/ Å	11.3054(2)
b/ Å	8.22080(10)
c/ Å	16.9376(5)
α/ °	90
β/ °	105.506(2)
γ/ °	90
V/ Å^3	1516.87(6)
ρ_{calcd}/g cm^{-1}	1.319
Z	4
T/K	100.00(10)
μ/mm^{-1}	0.093
F (000)	640.0
θ range/°	3.9 to 101.984
Reflections collected	56792
Independent reflections	13473
Data / restraints / parameters	13473/0/207
R_{int}	0.0232
Final R_1 values (I > 2σ(I))	0.0452
Final R_1 values (all data)	0.0684
wR_2 (all data)	0.1470
Goodness-of-fit on F^2	1.038

5. Fourier-transform infrared spectroscopy

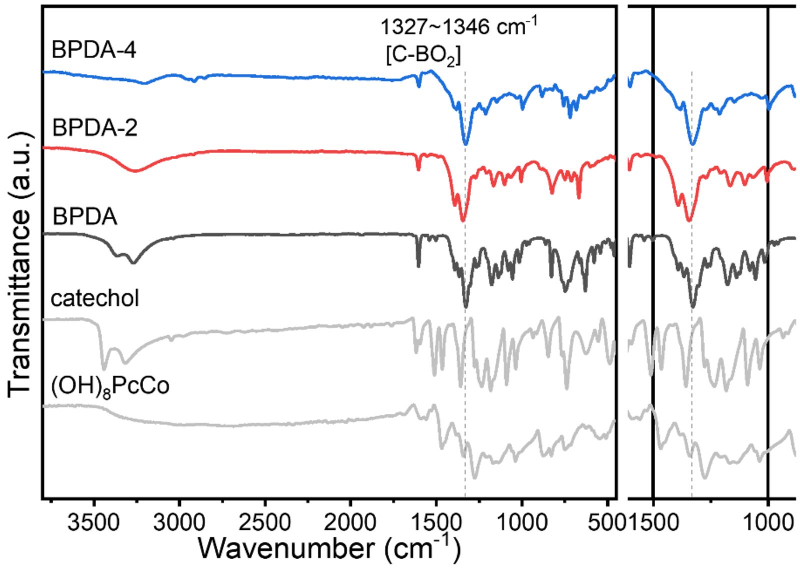

Figure S11. FTIR spectra of the precursors. Signals between 1327 ~ 1346 cm^{-1} corresponds to B-O stretching vibrations of the trigonal [-BO_2].[9]

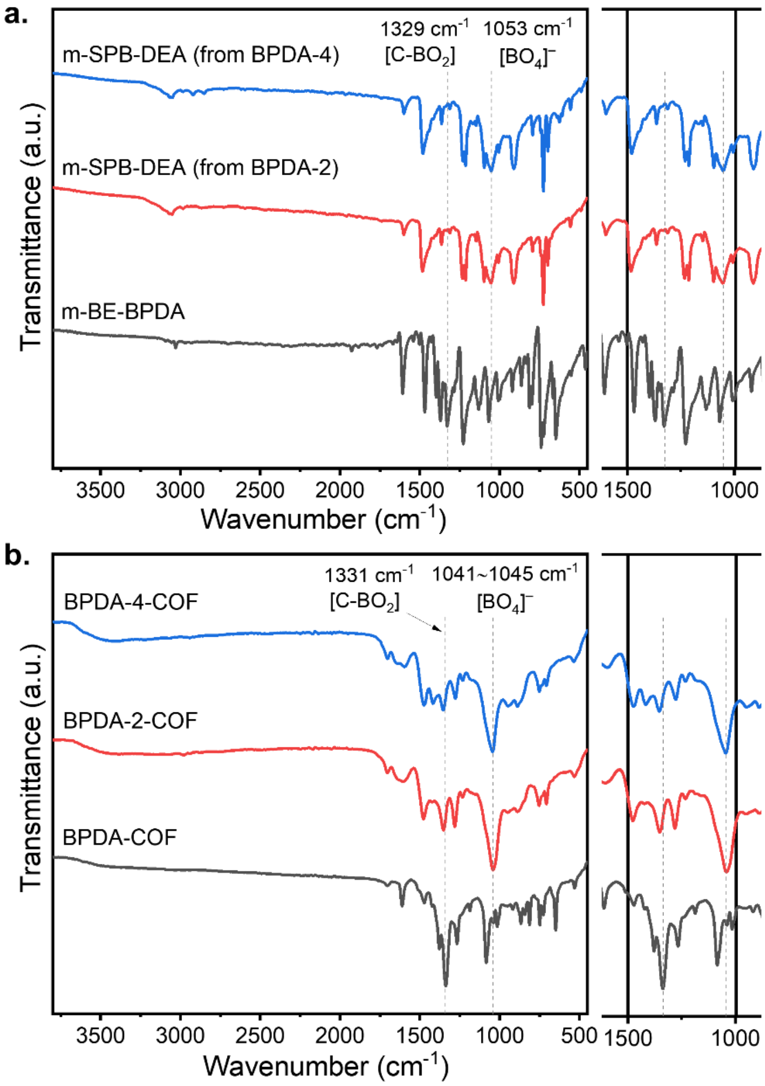

Figure S12. (a) FTIR spectra of the model compounds isolated from the reaction between catechol with linker **BPDA**, **BPDA-2** and **BPDA-4**, respectively. **(b)** FTIR spectra of the **BPDA-COF**, **BPDA-2-COF** and **BPDA-4-COF**.

6. Solid state NMR spectra

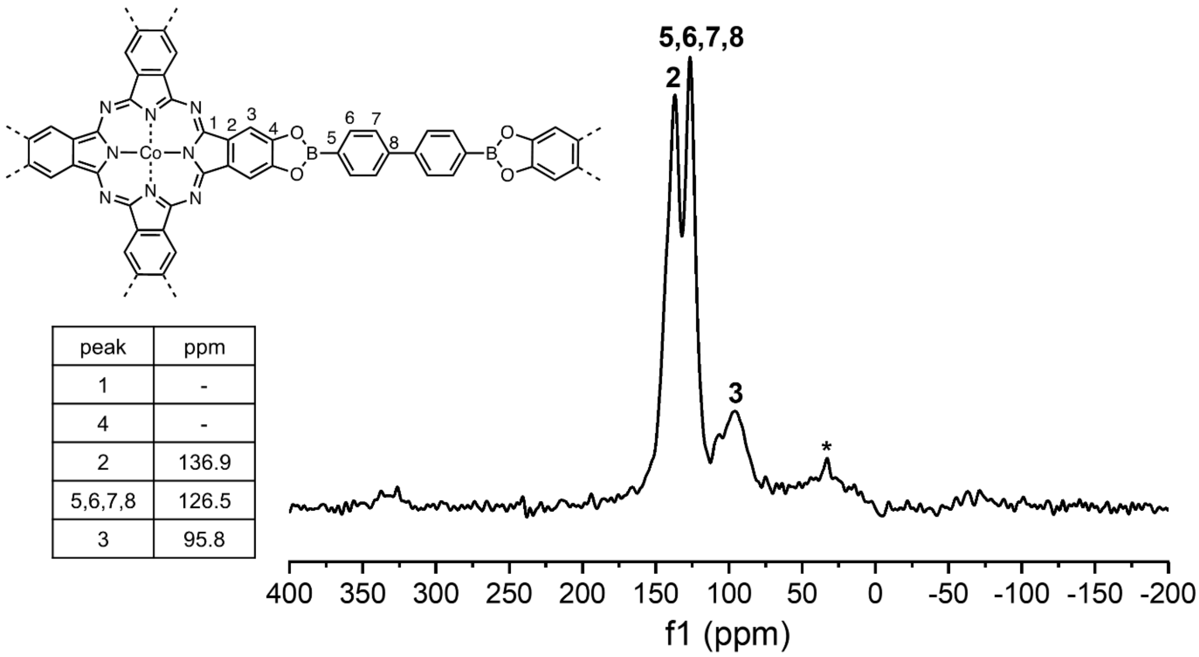

peak	ppm
1	-
4	-
2	136.9
5,6,7,8	126.5
3	95.8

Figure S13. Solid state ^{13}C CP/MAS NMR spectrum of the 2D **BPDA-COF**. Supercritical CO_2 activated COF was used for the measurement. *Signal marked by asterisk at ~ 33.0 ppm is supposed to be the residue solvent.

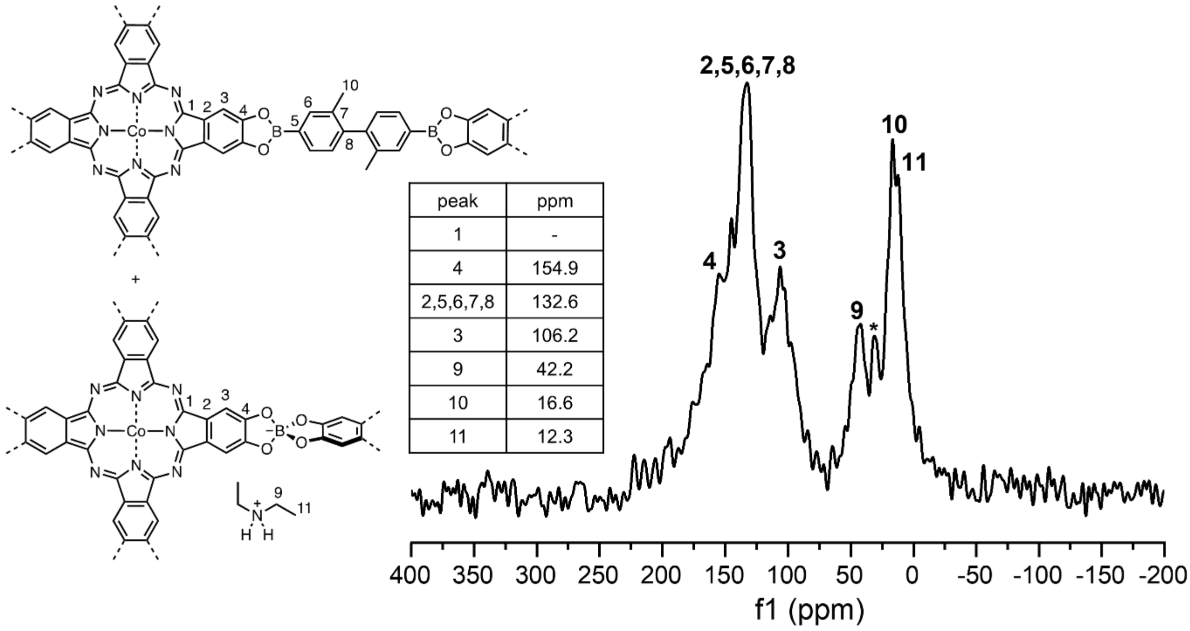

peak	ppm
1	-
4	154.9
2,5,6,7,8	132.6
3	106.2
9	42.2
10	16.6
11	12.3

Figure S14. Solid state ^{13}C CP/MAS NMR spectrum of the **BPDA-2-COF** of mixed 2D boronate ester and 3D spiroborate structures. Supercritical CO_2 activated COF was used for the measurement. *Signal marked by asterisk at ~ 31.0 ppm is supposed to be the residue solvent. For reference, the carbon signal of the methyl group on linker **BPDA-2** locates at around 20.0 ppm (**Figure S33**); the secondary and the primary carbon of ethyl group in $[NH_2Et_2]^+$ locates at 41.38 and 11.05 ppm (**Figure S3**).

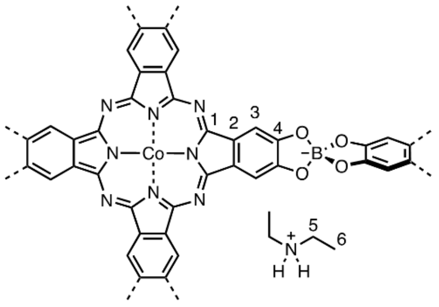

peak	ppm
1	-
4	150.8
2	134.0
3	107.1
5	43.6
6	13.7

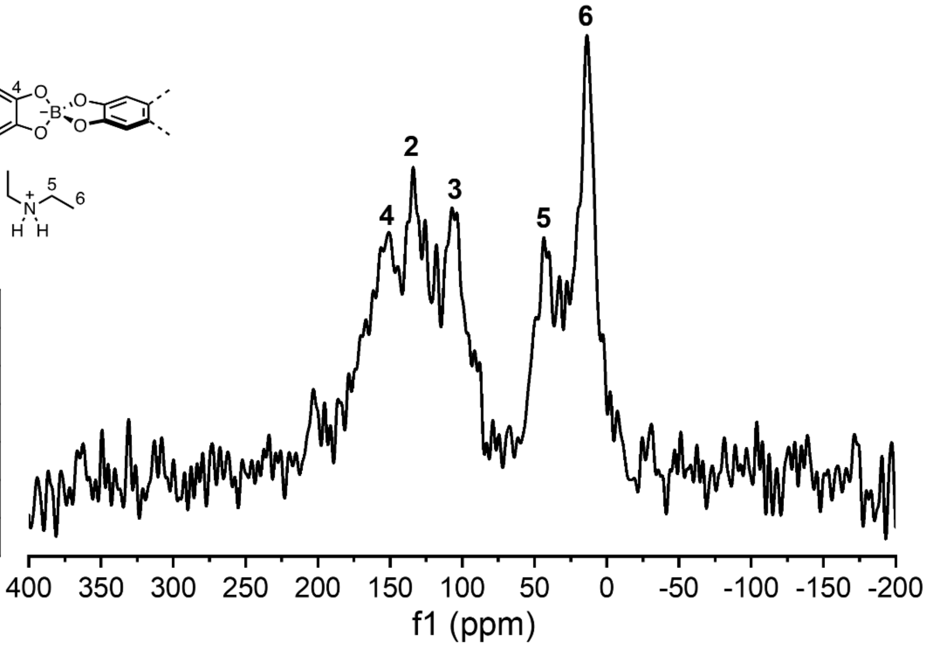

Figure S15. Solid state ^{13}C CP/MAS NMR spectrum of the **BPDA-4-COF** of 3D spiroborate structure. Supercritical CO_2 activated COF was used for the measurement. For reference, the secondary and the primary carbon of ethyl group in $[NH_2Et_2]^+$ locates at 41.38 and 11.09 ppm (**Figure S6**).

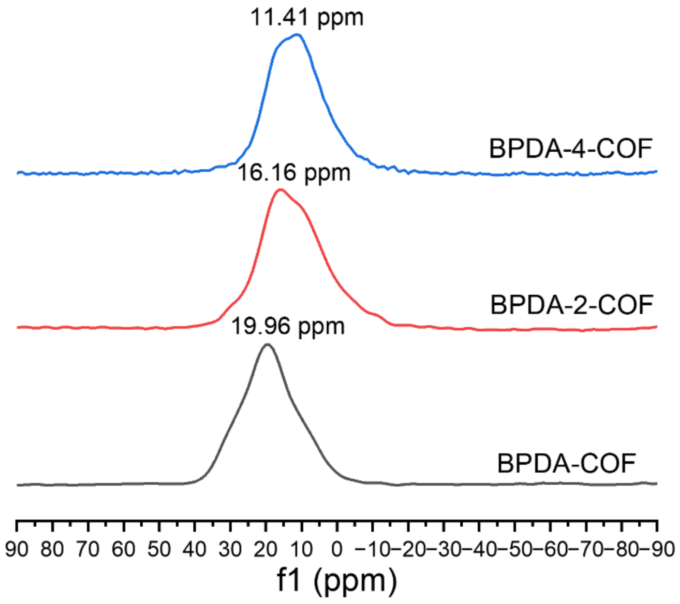

Figure S16. Solid-state ^{11}B MAS NMR spectrum of **BPDA-COF**, **BPDA-2-COF** and **BPDA-4-COF**. Supercritical CO_2 activated COF was used for the measurement.

7. Thermogravimetric analysis

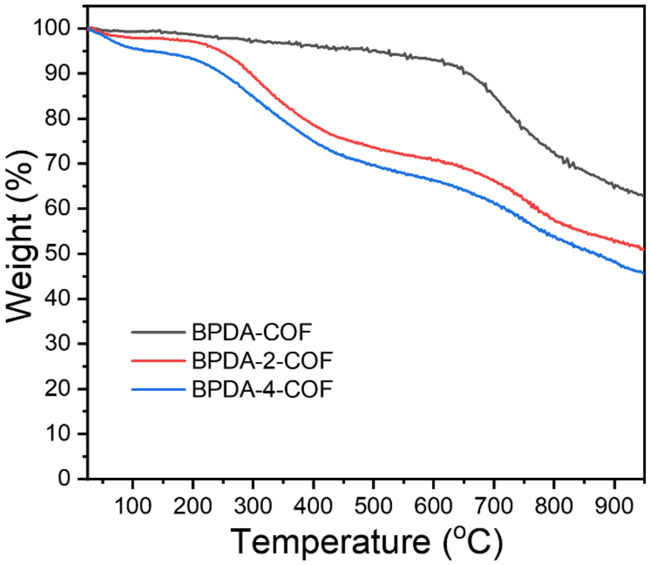

Figure S17. TGA curves of **BPDA-COF**, **BPDA-2-COF** and **BPDA-4-COF**. Supercritical CO_2 activated COF was used for the measurement.

8. Gas sorption isotherms

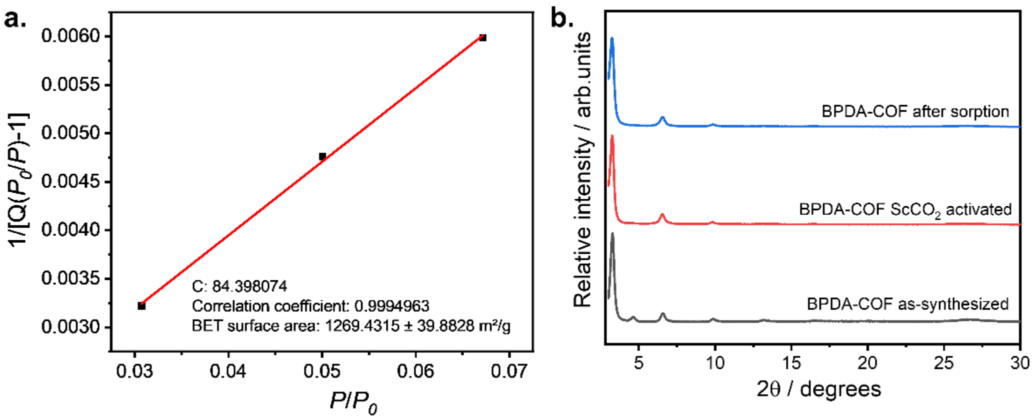

Figure S18. (a) BET surface area plot for **BPDA-COF**. **(b)** PXRD comparison of **BPDA-COF**, as synthesized, after $ScCO_2$ activation and after sorption test. We used $ScCO_2$ activated samples for sorption measurement.

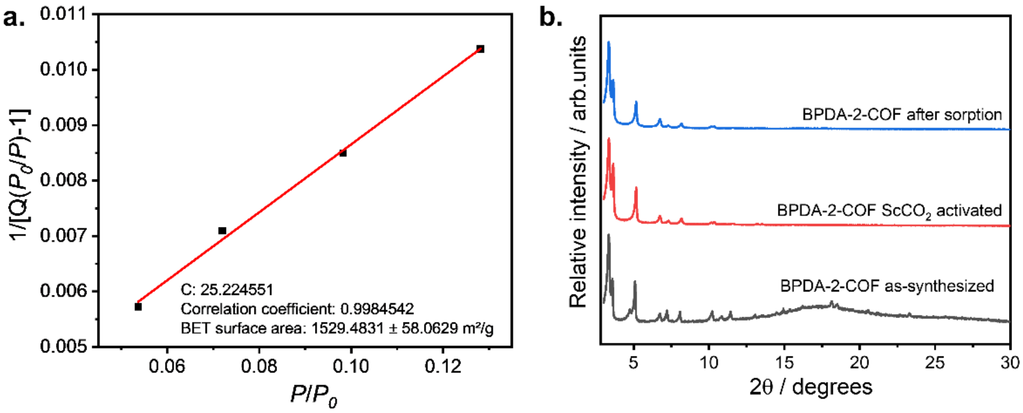

Figure S19. (a) BET surface area plot for **BPDA-2-COF**. **(b)** PXRD comparison of **BPDA-2-COF**, as synthesized, after $ScCO_2$ activation and after sorption test. We used $ScCO_2$ activated samples for sorption measurement. Unless otherwise specified, this batch of COF was used for all the characterizations.

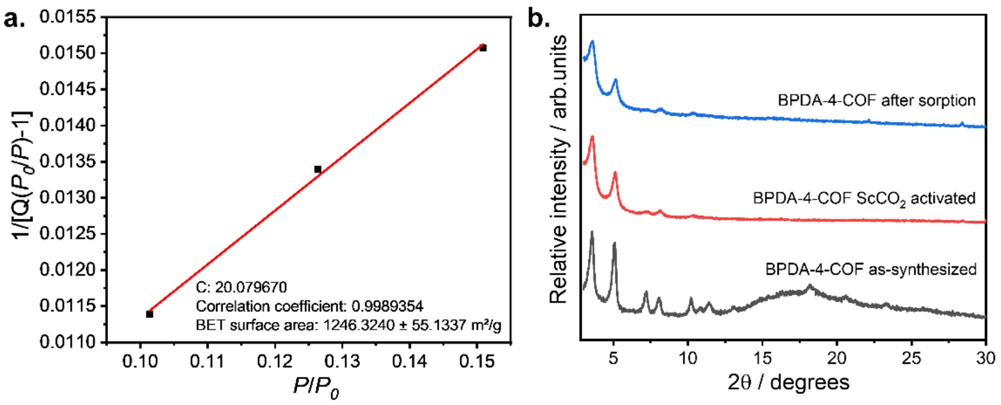

Figure S20. (a) BET surface area plot for **BPDA-4-COF**. **(b)** PXRD comparison of **BPDA-4-COF**, as synthesized, after $ScCO_2$ activation and after sorption test. We used $ScCO_2$ activated samples for sorption measurement.

9. Scanning electron microscopy

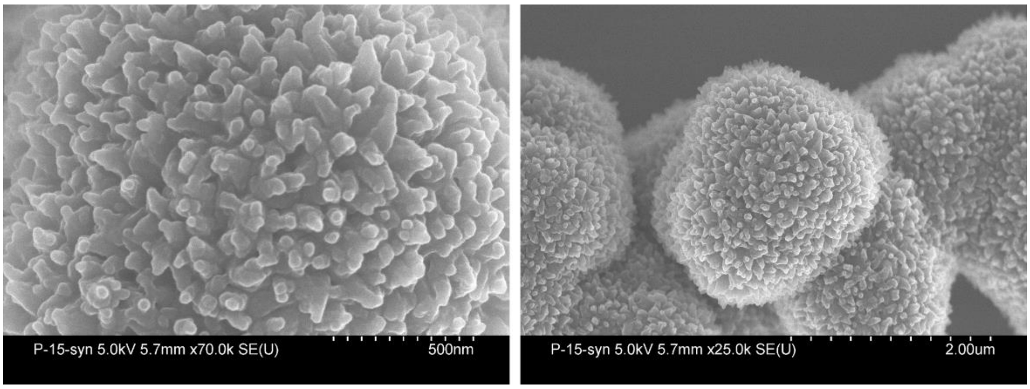

Figure S21. SEM images of **BPDA-COF**. As-synthesized COF material was used for measurement.

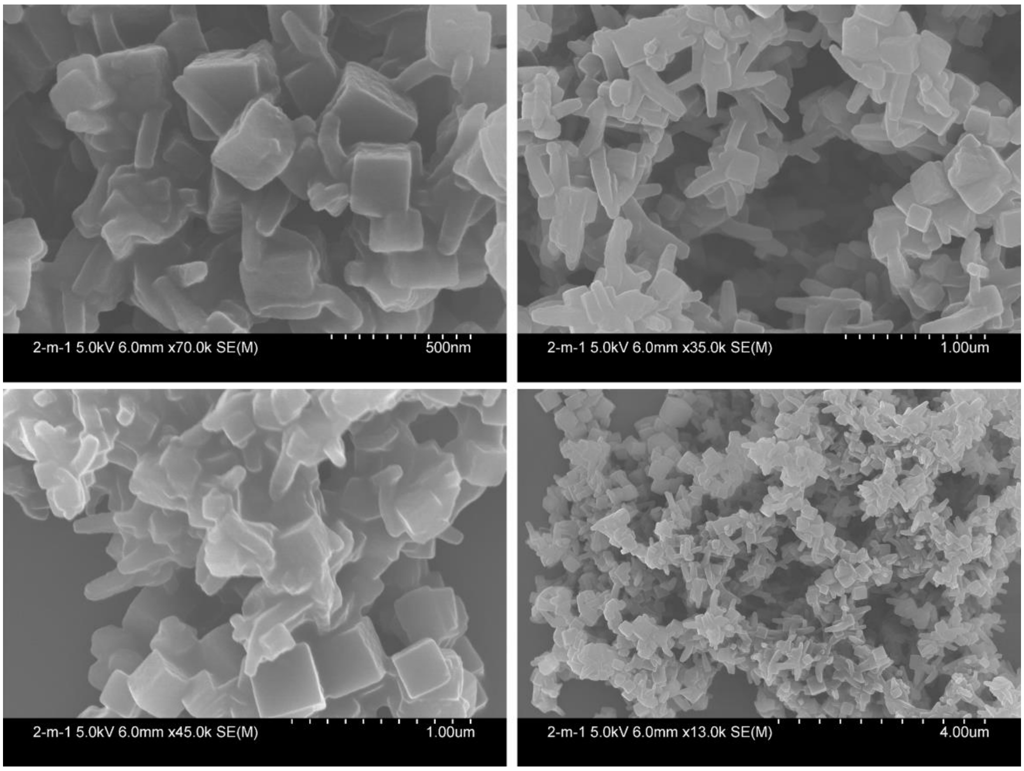

Figure S22. SEM images of **BPDA-2-COF**. As-synthesized COF material was used for measurement.

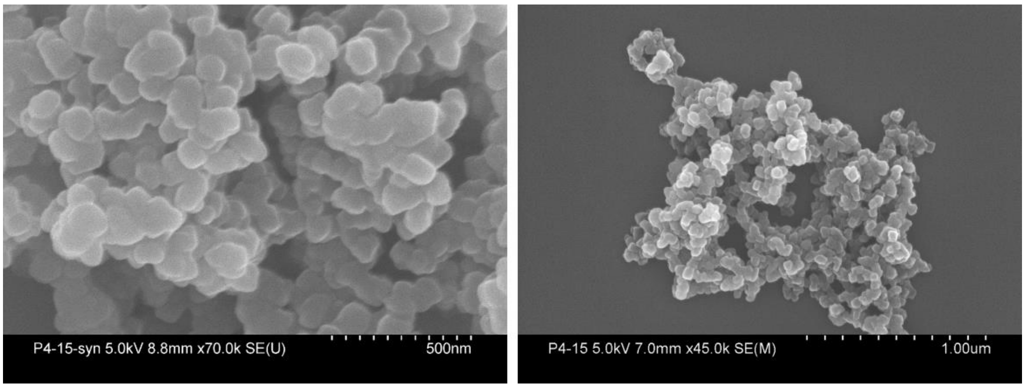

Figure S23. SEM images of **BPDA-4-COF**. As-synthesized COF material was used for measurement.

10. Transmission electron microscopy

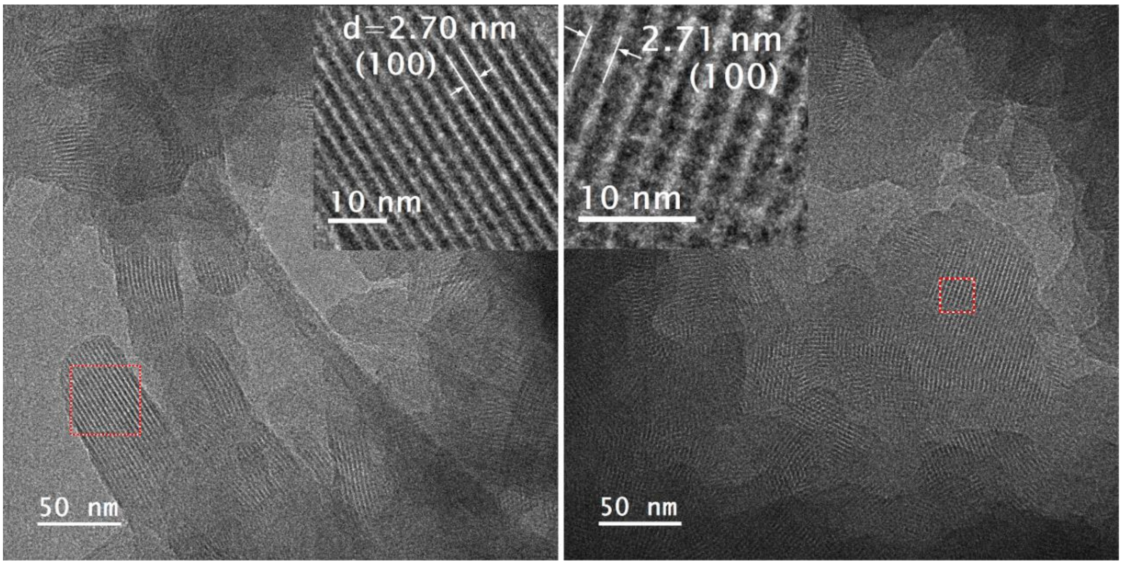

Figure S24. TEM images of **BPDA-COF**. As-synthesized COF material was used for measurement.

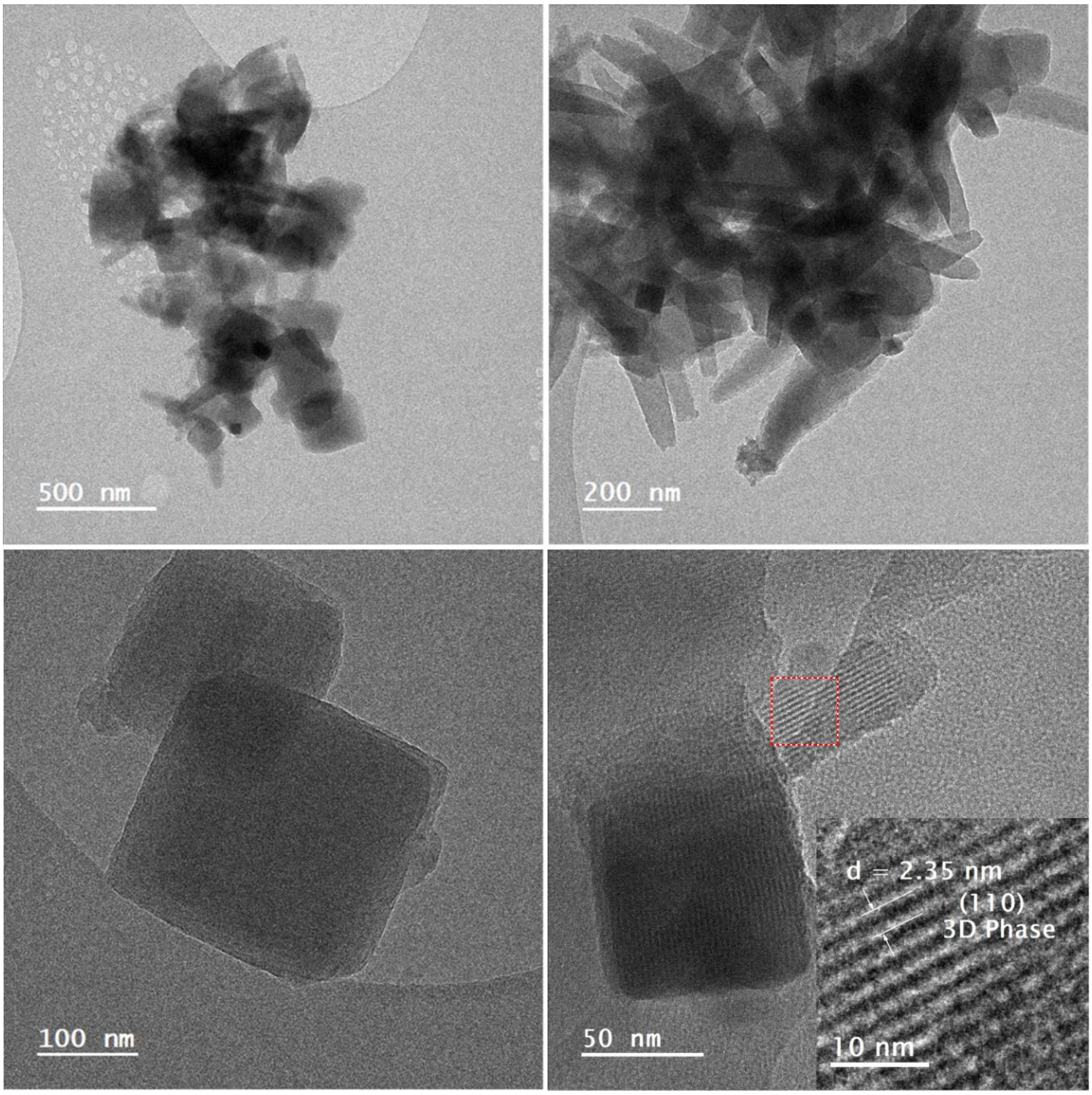

Figure S25. TEM images of **BPDA-2-COF**. As-synthesized COF material was used for measurement.

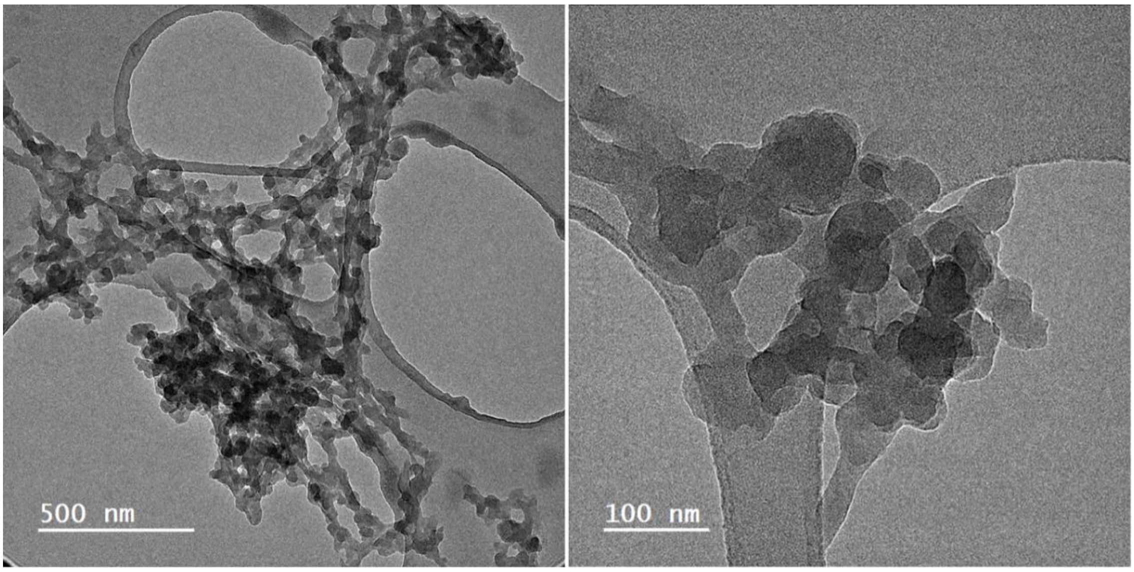

Figure S26. TEM images of **BPDA-4-COF**. As-synthesized COF material was used for measurement.

11. Structural modelling

Structural models of all COFs were constructed using zeo++[10] and Accelrys Materials Studio (version 5.5) program suite. All the geometry optimizations made use of the universal force field, implemented in the Forcite module of the BIOVIA Materials Studio software. The experimental PXRD data was refined by Pawley refinement in TOPAS Academic (**Figure 1b-d**).

Experimental PXRD of **BPDA-COF** fit well with previously reported COF in AA-stacking mode, we therefore did not construct another model in AB-stacking mode.[11, 12] For the 2D boronate ester phase of the mixed phase **BPDA-2-COF**, crystal models in both AA- and AB-stacking mode was constructed. The experimental PXRD fit well with the simulated pattern from COF model in AA-stacking mode (**Figure S27**). For the 3D phase of the mixed phase **BPDA-2-COF** and the 3D **BPDA-4-COF**, as their experimental PXRD pattern fit well with the reported 3D spiroborate COF of non-interpenetrated **nbo** topology, we then used the same structural model as reports.[2, 12]

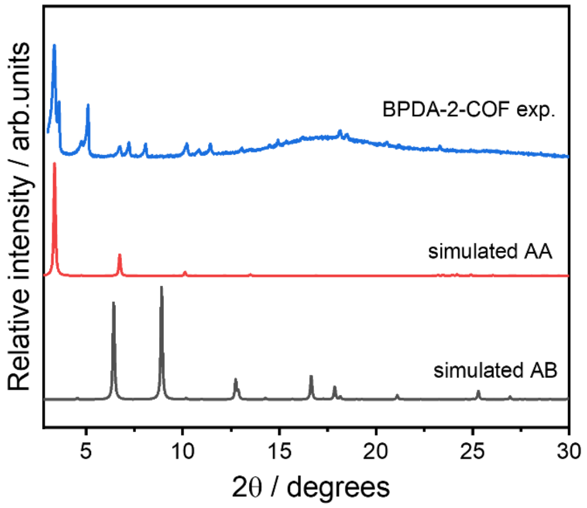

Figure S27. PXRD comparison between the experimental pattern of **BPDA-2-COF** and the simulated pattern based-on the corresponding 2D boronate ester COF model in AA- and AB-stacking mode. PXRD comparison result shows that the 2D phase within **BPDA-2-COF** is of AA-stacking mode.

Table S2. Atomic coordinates and unit cell parameters for the 2D **BPDA-COF** in AA-stacking mode.

**BPDA-COF**, AA-stacking mode				
Space group: P4/MMM (No.123)<br>a = b = 26.9139 Å, c = 3.5909 Å				
Atom Name	x	y	z	Occupancy
H	0.69473	0.40726	0.5	1
C	0.6519	0.47479	0.5	1
C	0.60111	0.46034	0.5	1
C	0.69491	0.44714	0.5	1
C	0.73841	0.47431	0.5	1
O	0.78492	0.45455	0.5	1
C	0.89466	0.45547	0.5	1
C	0.94563	0.45533	0.5	1
H	0.87519	0.42057	0.5	1
H	0.9624	0.41946	0.5	1
N	0.58666	0.41334	0.5	1
N	0.57092	0.5	0.5	1
B	0.81345	0.5	0.5	1
C	0.86941	0.5	0.5	1
C	0.9725	0.5	0.5	1
Co	0.5	0.5	0.5	1

Table S3. Atomic coordinates and unit cell parameters for the 2D boronate ester phase in **BPDA-2-COF** in AA-stacking mode.

the 2D boronate ester phase in **BPDA-2-COF**, AA-stacking mode				
Space group: P4 (No.75)<br>a = b = 26.0912 Å, c = 3.8144 Å				
Atom Name	x	y	z	Occupancy
H	0.6974	0.40589	0.27674	1
C	0.65762	0.47469	0.11654	1
C	0.60535	0.45943	0.05484	1
C	0.69886	0.44689	0.24677	1
C	0.74079	0.4754	0.35992	1
O	0.7838	0.45576	0.51748	1
C	0.8873	0.4599	0.88527	1
C	0.93891	0.45818	0.98167	1
H	0.86357	0.42642	0.92149	1
C	0.95622	0.40914	1.14553	1
H	0.69787	0.59704	0.23102	1
C	0.65754	0.52695	0.10754	1
C	0.60507	0.54165	0.04677	1
C	0.69909	0.55583	0.22096	1
C	0.74111	0.52837	0.34387	1
O	0.78473	0.54948	0.48363	1
C	0.89608	0.5472	0.70353	1
C	0.94754	0.54579	0.78763	1
H	0.88041	0.58177	0.59273	1
H	0.96877	0.57978	0.73383	1
N	0.59001	0.41082	0.07319	1
N	0.5741	0.50036	0.02067	1
B	0.81114	0.5031	0.59416	1
C	0.86637	0.50365	0.73817	1
C	0.97116	0.50125	0.91622	1
H	0.95481	0.37778	0.95317	1
H	0.93045	0.39893	1.36165	1
H	0.99502	0.41164	1.2491	1
Co	0.5	0.5	0.01721	1

Table S4. Atomic coordinates and unit cell parameters for the 2D boronate ester phase in **BPDA-2-COF** in AB-stacking mode.

**BPDA-2-COF**, AB-stacking mode				
Space group: I4 (No. 79)<br>a = b = 27.4821 Å, c = 10.6411 Å				
Atom Name	x	y	z	Occupancy
H	1.19423	-0.09184	0.27061	1
C	1.15145	-0.02535	0.2602	1
C	1.10052	-0.03962	0.26011	1
C	1.19449	-0.05253	0.26493	1
C	1.23806	-0.02589	0.26203	1
O	1.28447	-0.04539	0.26705	1
C	1.39613	-0.03986	0.30901	1
C	1.44729	-0.04002	0.31029	1
H	1.37633	-0.06962	0.35218	1
C	1.47337	-0.08111	0.37565	1
H	0.80528	-0.09077	0.24415	1
C	0.84842	-0.02453	0.25521	1
C	0.89926	-0.03907	0.2555	1
C	0.80523	-0.05147	0.24997	1
C	0.76181	-0.02459	0.25242	1
O	0.7153	-0.04382	0.24659	1
C	0.60412	-0.03748	0.20044	1
C	0.5534	-0.03681	0.19637	1
H	0.62339	-0.06765	0.15778	1
H	0.53463	-0.06602	0.14829	1
N	1.08569	-0.08616	0.25776	1
N	1.07072	-0.00019	0.25786	1
B	1.31358	-0.00087	0.25659	1
C	1.37042	-0.00106	0.25544	1
C	1.47296	-0.0011	0.25299	1
H	1.49188	-0.10467	0.30601	1
H	1.44767	-0.10448	0.42884	1
H	0.50055	-0.06636	0.44279	1
Co	1	0	0.25786	1

Table S5. Atomic coordinates and unit cell parameters for the 3D **BPDA-4-COF**.

**BPDA-4-COF**, 3D non-interpenetrated nbo topoloy				
Space group: IM-3M (No.229)<br>a = b = c = 34.7246 Å				
Atom Name	x	y	z	Occupancy
C	0.02028	0.11891	0.5	1
C	0.96798	0.07884	0.5	1
C	0.15302	0.95804	0.5	1
C	0.18662	0.97954	0.5	1
O	0.96592	0.77631	0.5	1
H	0.92667	0.84711	0.5	1
N	0.06867	0.06867	0.5	1
N	0	0.05478	0.5	1
B	0	0.75	0.5	1
Co	0	0.5	0.5	1

12. Mechanistic study

General way of reacting linkers in DEF: A 10 mL Pyrex tube was charged with diboronic acid linker (30.0 mg) and *N*,*N*-diethylformamide (DEF) (0.5 mL). The mixture was sonicated at room temperature for 2 minutes, then flash frozen in a liquid N_2 bath and degassed through three freeze-pump-thaw cycles and sealed under vacuum using a Schlenk line and oil pump. Upon warming to room temperature, the tube was put into a 120 °C oven for 72 hours and then taken out of the oven, which produced a transparent colourless solution (with very trace amount of white precipitates in, supposed to be the trimerized product of either boric acid or boronic acid, did not characterize in detail here). After cooling to room temperature, 0.25 mL reaction solution was mixed with 0.25 mL dmso-d_6 for solution ^{11}B NMR measurement. Solution ^{11}B NMR measurements were conducted in quartz NMR tube.

General way of reacting linkers in neutral condition: The reaction was conducted following the above procedure, but replacing DEF by 0.5 mL solvent mixture of 1,4-dioxane : methanol = 2:1 (v:v).

Solution ^{11}B NMR measurements of the linker **BPDA**, **BPDA-2** and **BPDA-4** were conducted in quartz NMR tube, by dissolving 15 mg of each linker in 0.6 mL dmso-d_6.

Samples for LC-MS measurement were prepared at a concentration of 1 mg.mL^{-1} in methanol.

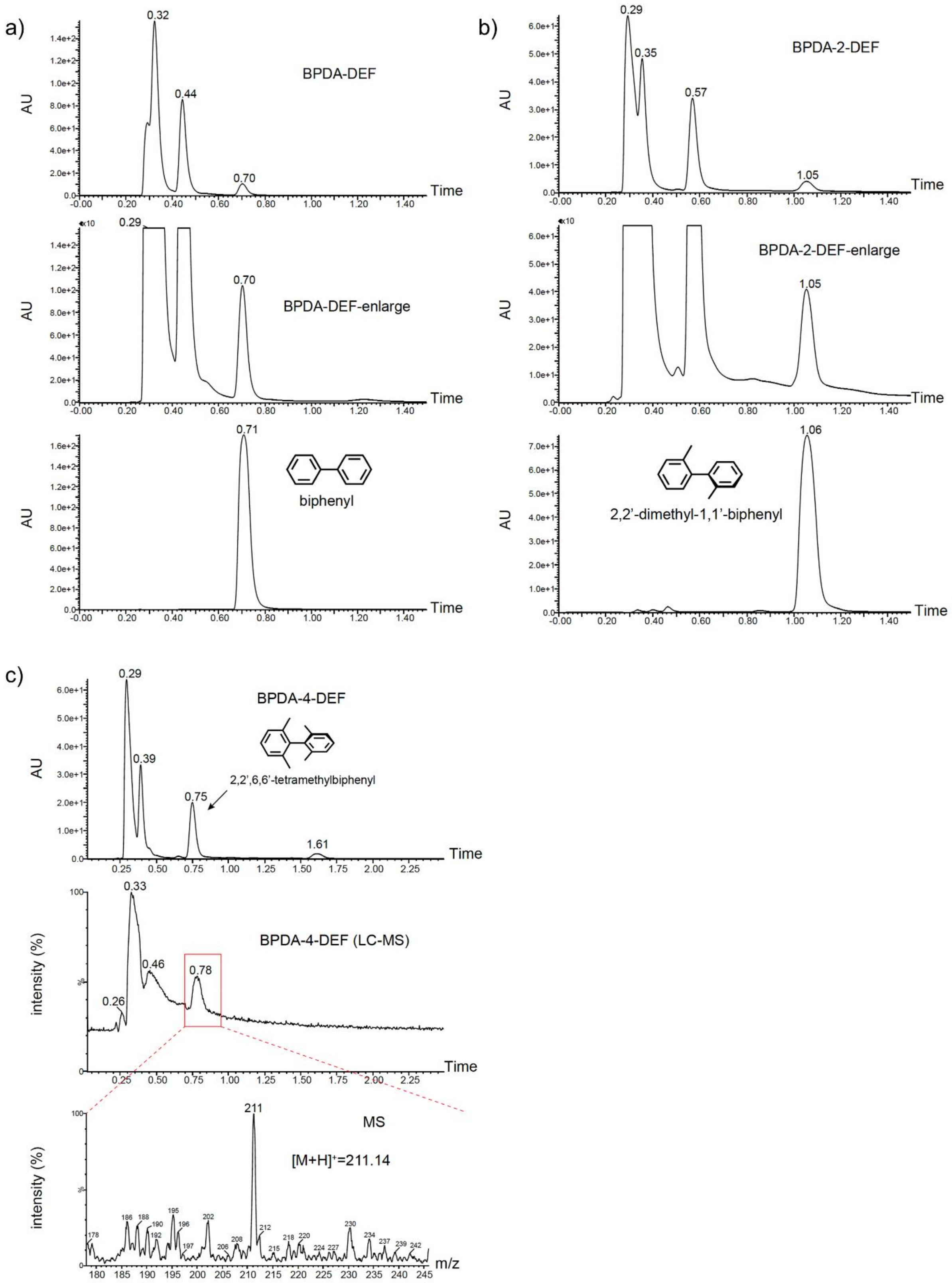

Figure S28. LC-MS of the reaction mixture of linker **(a) BPDA**, **(b) BPDA-2** and **(c) BPDA-4** after reacted in DEF at 120 ºC for 3 days, and their comparison with biphenyl and 2,2'-dimethyl-1,1'-biphenyl, respectively. LC-MS results confirmed the protodeboronation of linker **BPDA**, **BPDA-2** and **BPDA-4** in DEF.

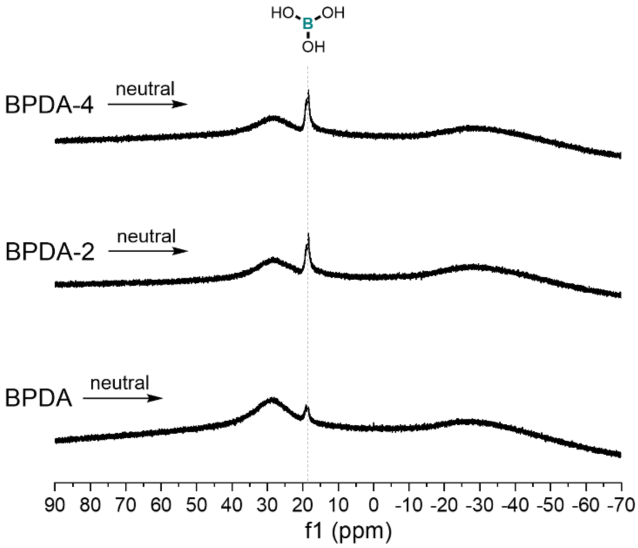

Figure S29. Solution ^{11}B NMR of the **BPDA**, **BPDA-2** and **BPDA-4** reaction mixture under neutral conditions, in dmso-d_6. Solution ^{11}B NMR conducted in quartz NMR tube, the broad signal at ~ 30.0 ppm is the background signal from quartz NMR tube. *Like the conclusion from our previous study, no formation of anionic $[B(OH)_4]^-$ under neutral condition.[12]

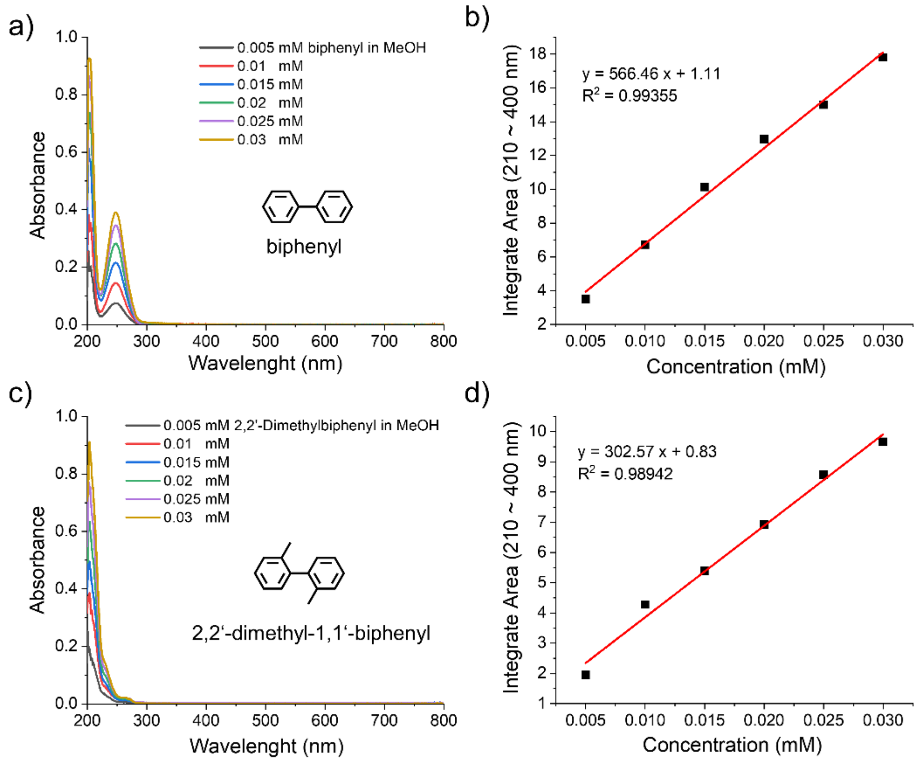

Figure S30. (a) (c) Absorption spectra of biphenyl and 2,2'-dimethyl-1,1'-biphenyl at different concentrations in MeOH. And, linear calibration plots of absorbance between 210 ~ 400 nm (integrate area) *vs.* the concentration of **(b)** biphenyl and **(d)** 2,2'-dimethyl-1,1'-biphenyl.

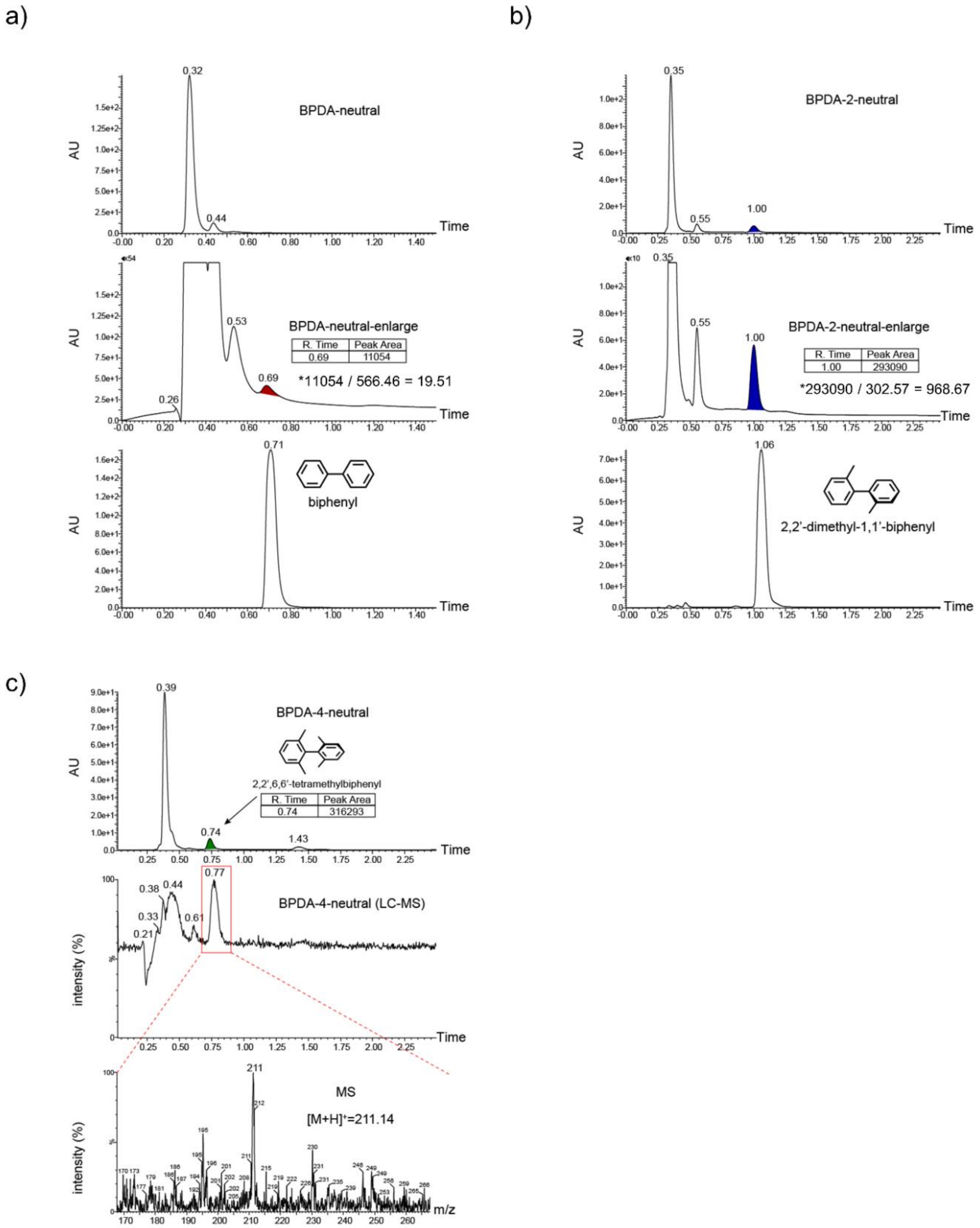

Figure S31. LC-MS of the reaction mixture of **(a) BPDA**, **(b) BPDA-2** and **(c) BPDA-4** after reacted in a neutral solvent system (1,4-dioxane: methanol = 2:1, v: v) at 120 ºC for 3 days, and their comparison with biphenyl and 2,2'-dimethyl-1,1'-biphenyl, respectively. LC-MS results confirmed the protodeboronation of **BPDA**, **BPDA-2** and **BPDA-4** under neutral condition. *As both samples for LC test were prepared at a concentration of 1 mg.mL^{-1} (1 μL was injected for each analysis), the higher relative concentration of 2,2'-dimethyl-1,1'-biphenyl to biphenyl (= 968.67/19.51) in their reaction mixture supports that linker **BPDA-2** is more liable to protodeboronation as compared to **BPDA** under neutral condition.

The relative concentration of biphenyl to 2,2'-dimethyl-1,1'-biphenyl was calculated based-on their calibration curve shown in **Figure S30** and the integrated peak area in LC-MS.

13. Solution NMR spectra of precursors

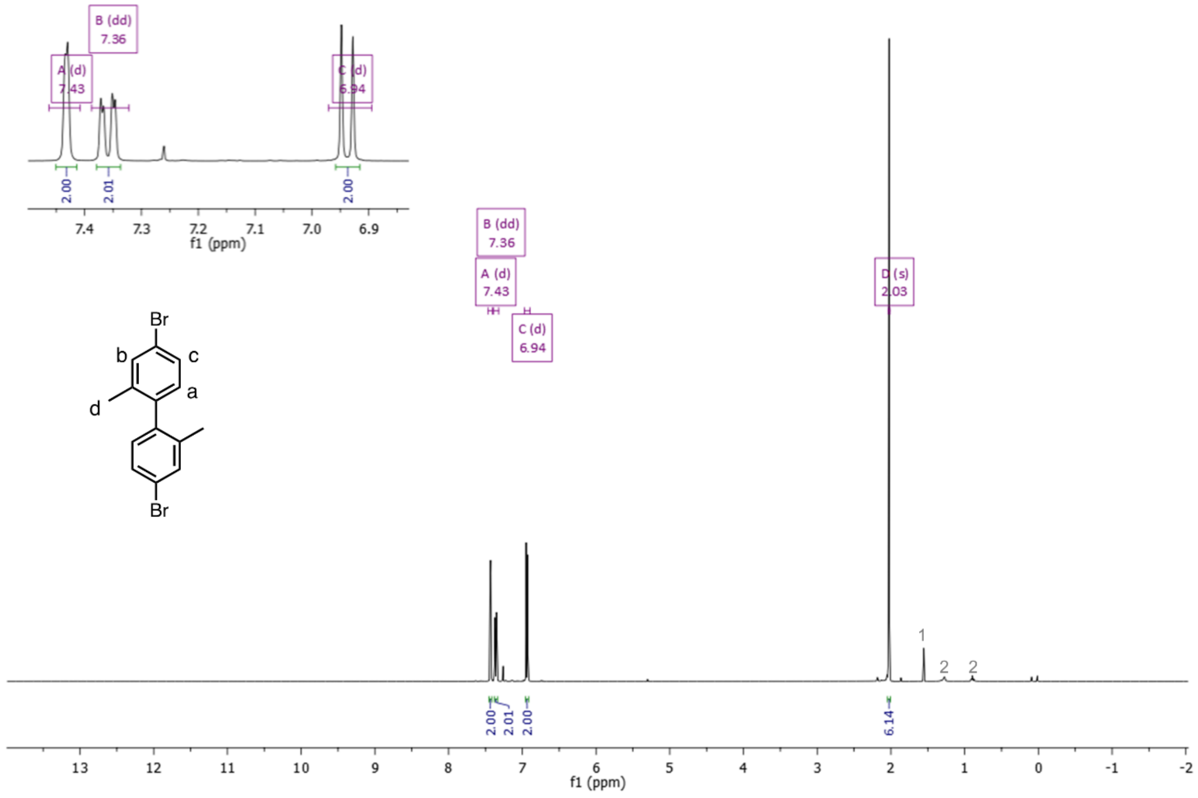

Figure S32. ^{1}H NMR spectrum of **BPDA-2-2Br** in $CDCl_3$. *1. H_2O (s, 1.55 ppm); 2. n-hexane (m, 1.28 ppm; t, 0.89 ppm).

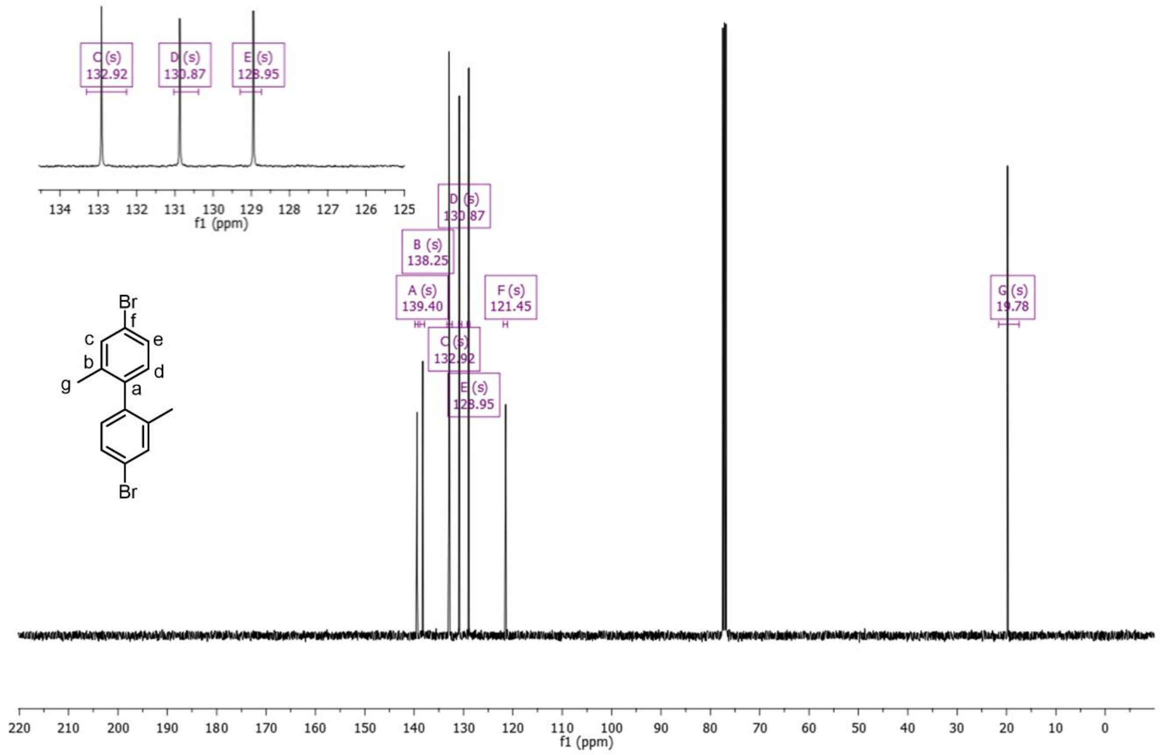

Figure S33. ^{13}C NMR spectrum of **BPDA-2-2Br** in $CDCl_3$.

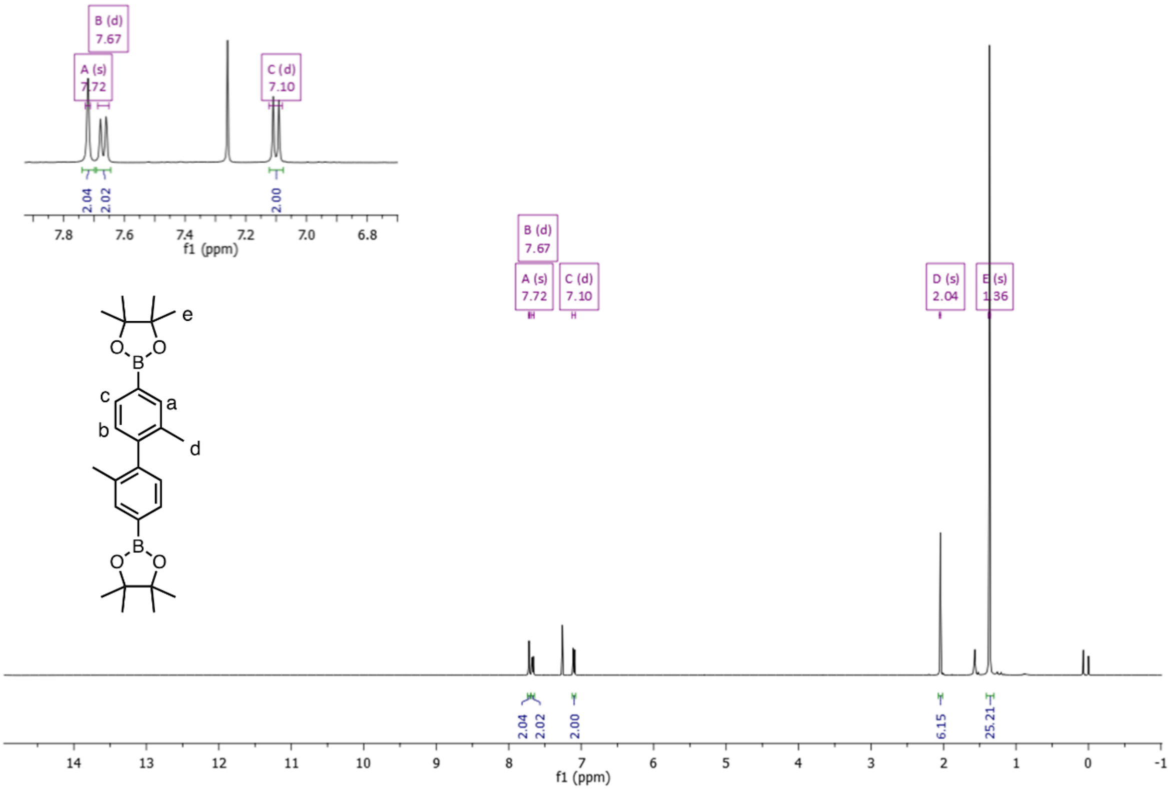

Figure S34. ^{1}H NMR spectrum of **BPDA-2-2Bpin** in $CDCl_3$.

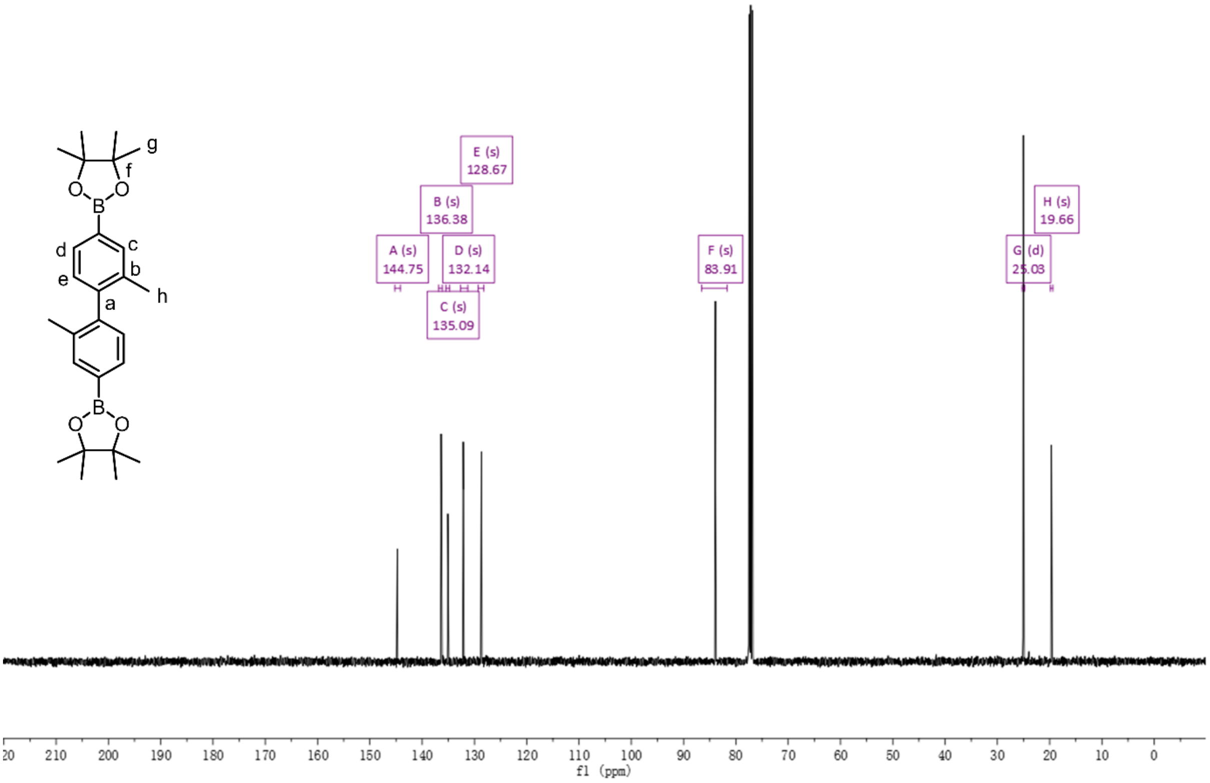

Figure S35. ^{13}C NMR spectrum of **BPDA-2-2Bpin** in $CDCl_3$.*C directly connected with B cannot be detected sometimes.

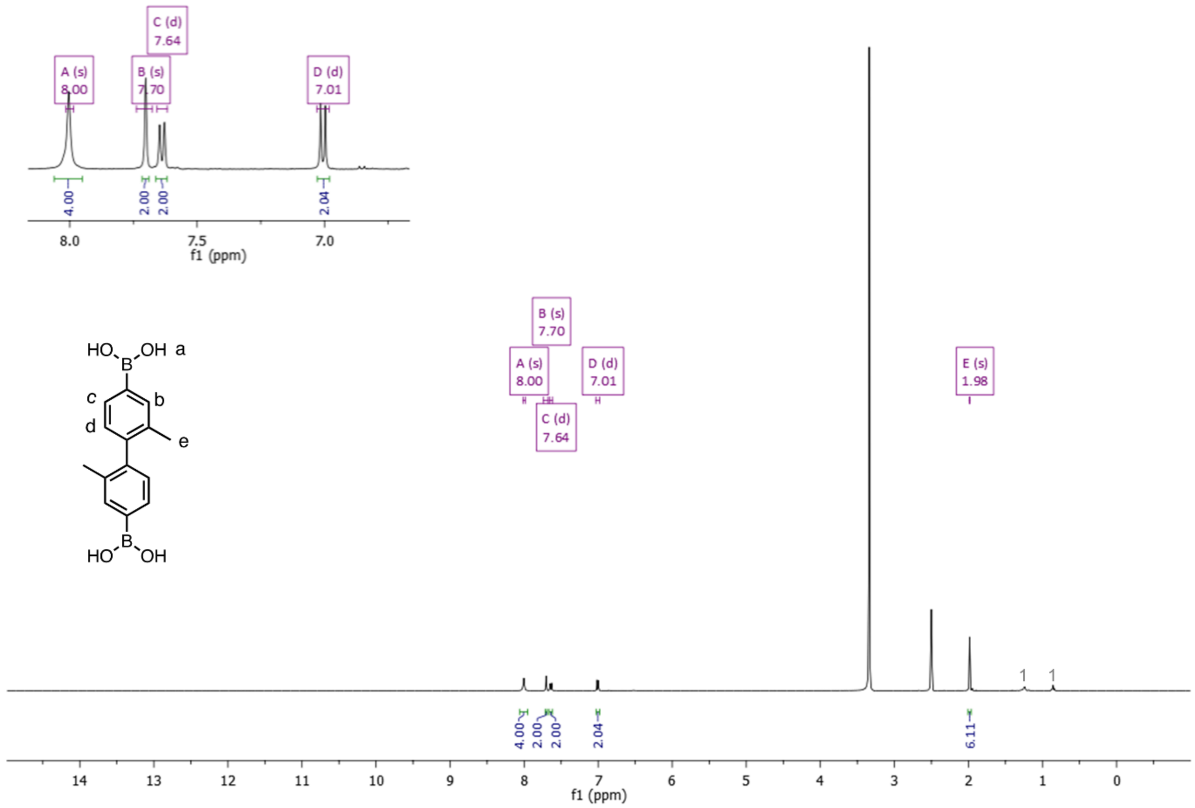

Figure S36. ^{1}H NMR spectrum of **BPDA-2** in dmso-d_6. *1. n-hexane (m, 1.24 ppm; t, 0.86 ppm).

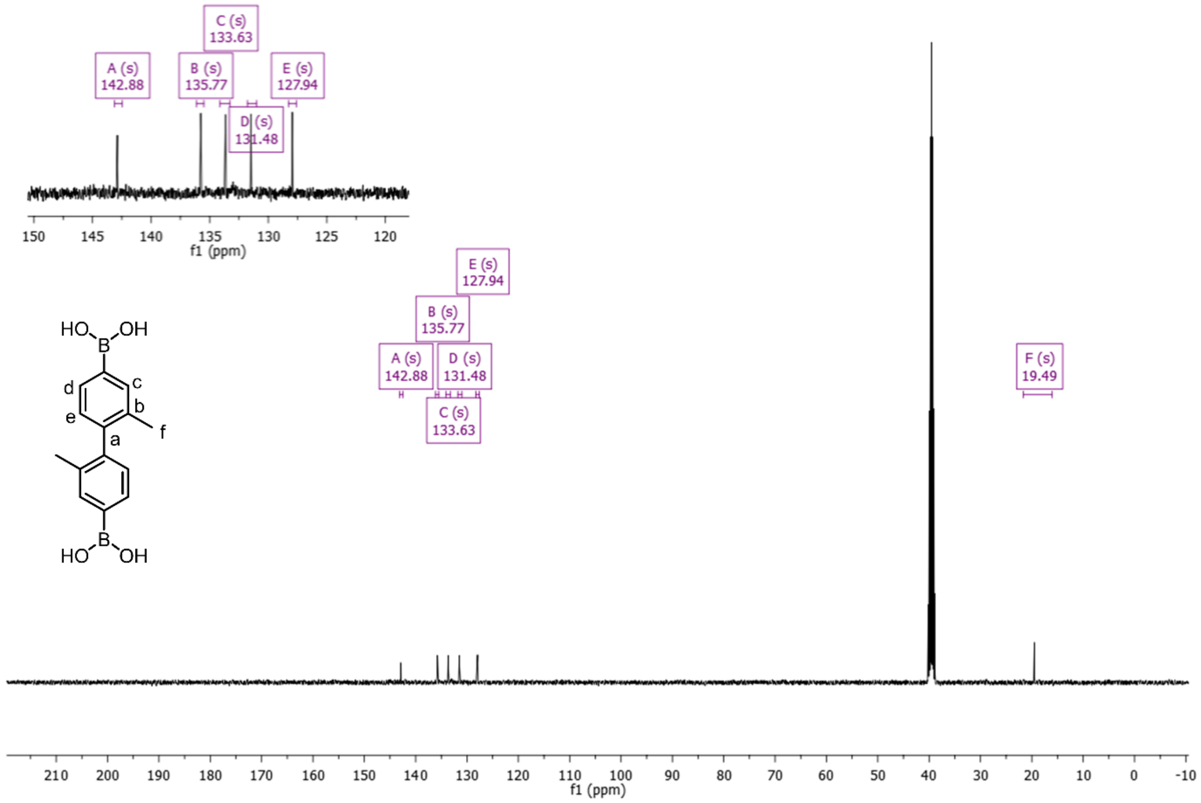

Figure S37. ^{13}C NMR spectrum of **BPDA-2** in dmso-d_6. *C directly connected with B cannot be detected sometimes.

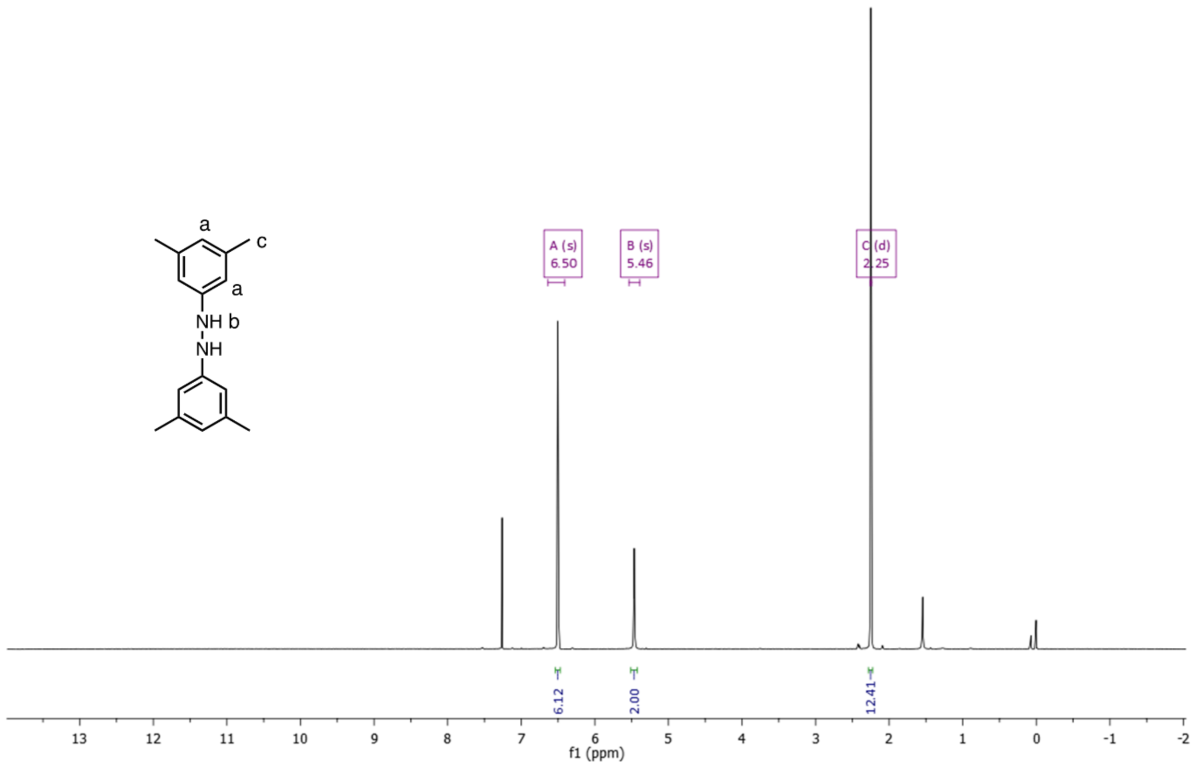

Figure S38. ^{1}H NMR spectrum of hydrazine in $CDCl_3$.

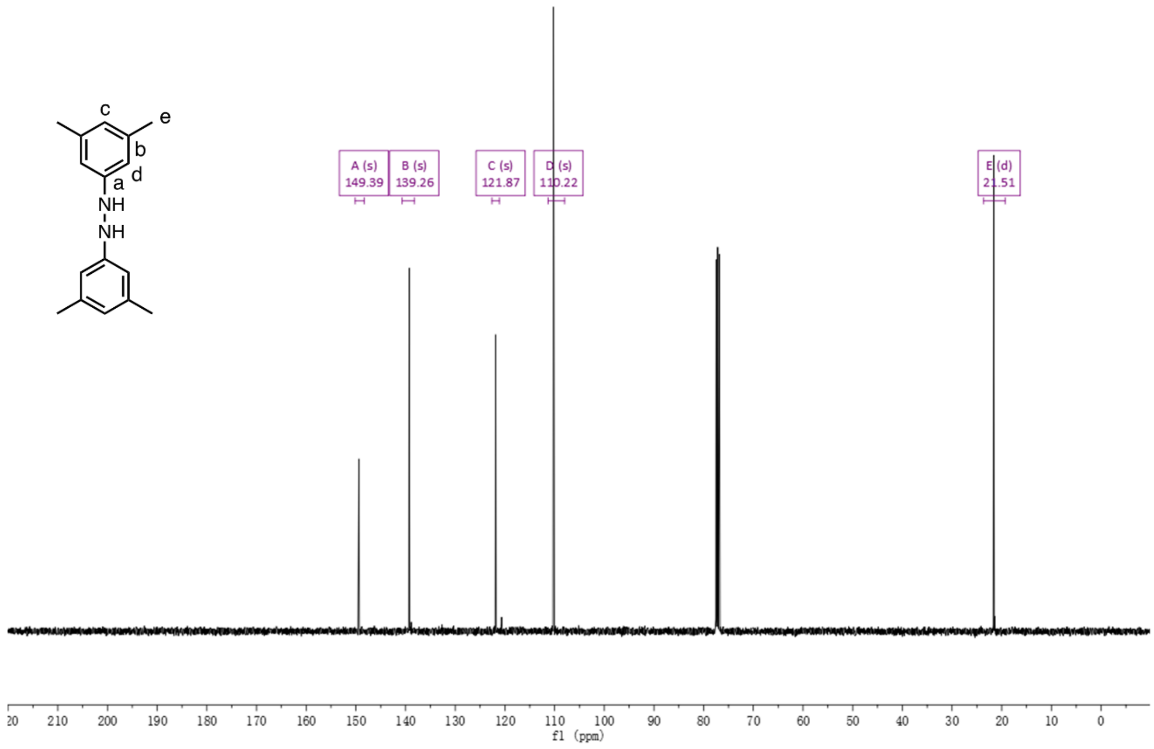

Figure S39. ^{13}C NMR spectrum of hydrazine in $CDCl_3$.

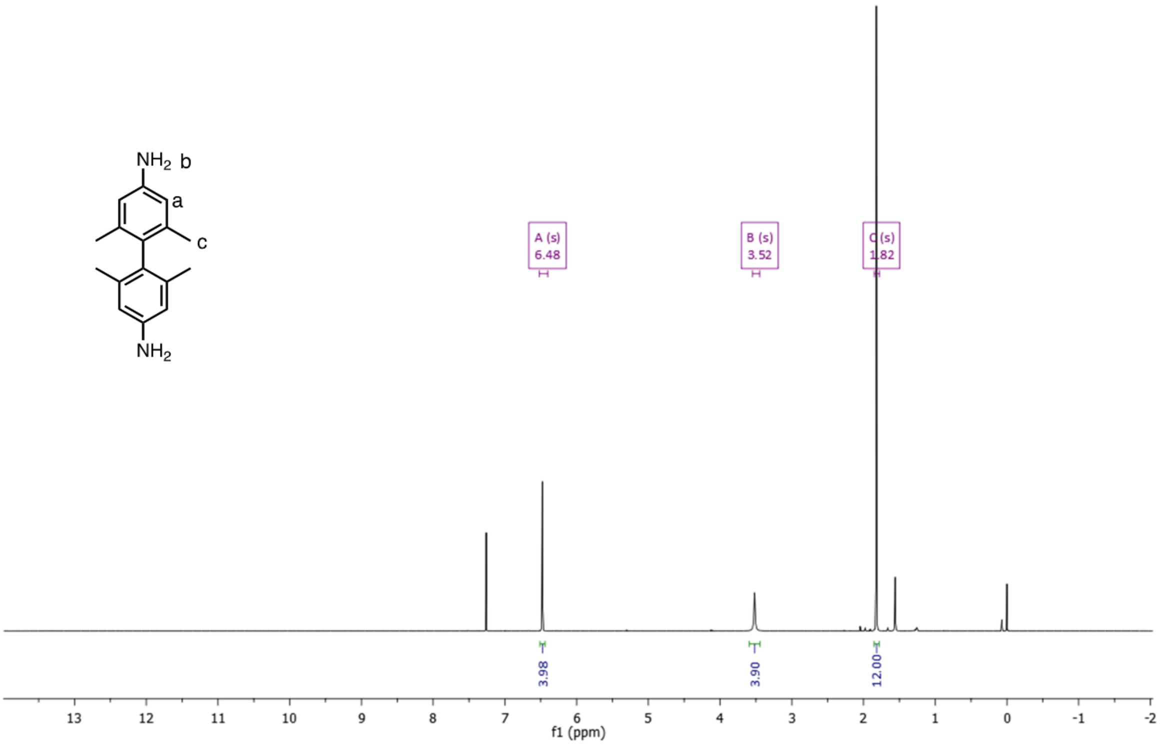

Figure S40. ^{1}H NMR spectrum of **BPDA-4-2NH$_2$** in $CDCl_3$.

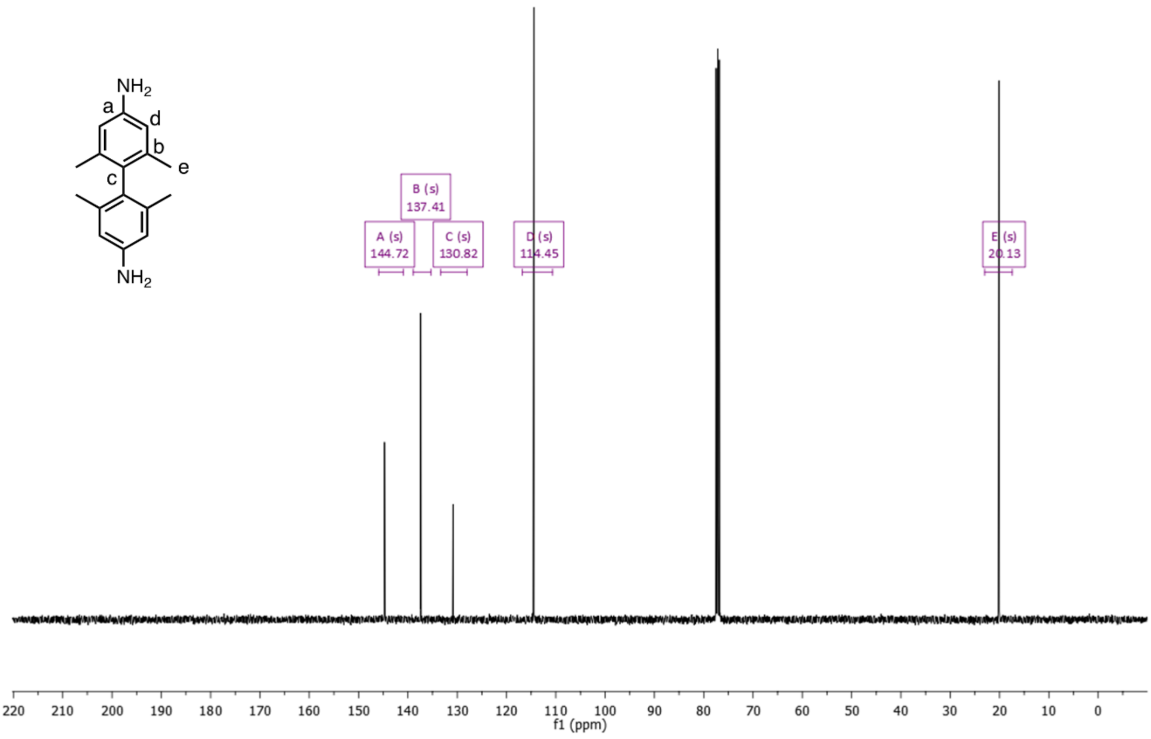

Figure S41. ^{13}C NMR spectrum of **BPDA-4-2NH$_2$** in $CDCl_3$.

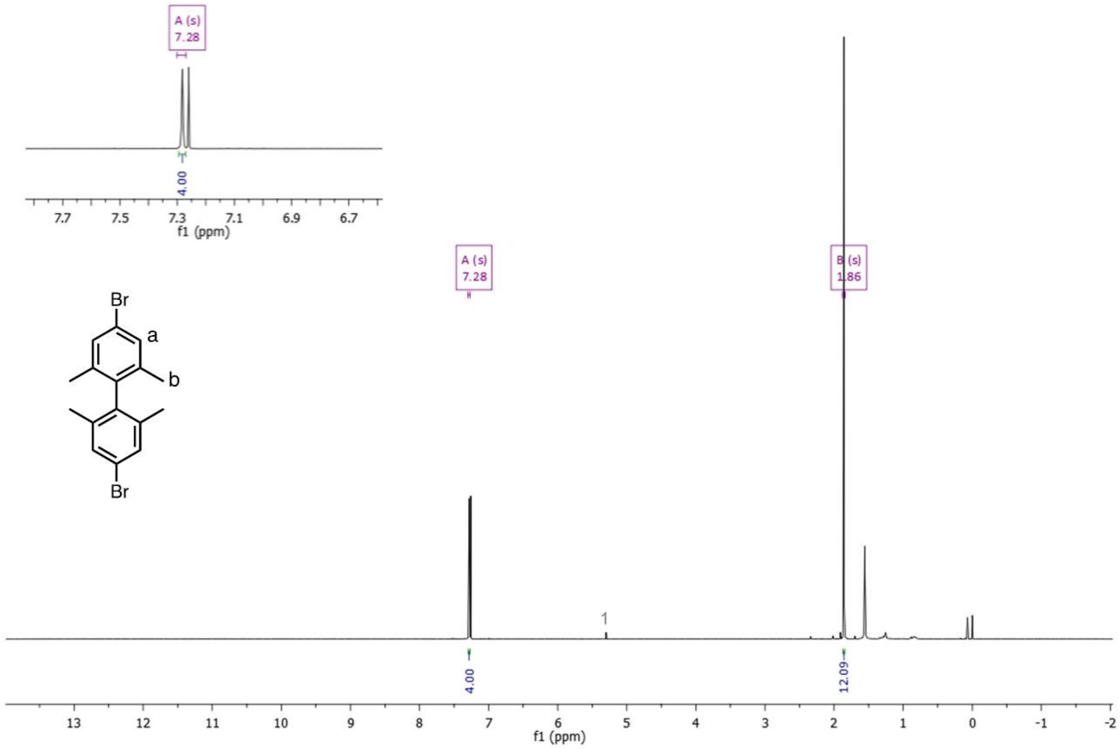

Figure S42. ^{1}H NMR spectrum of **BPDA-4-2Br** in $CDCl_3$. *1. CH_2Cl_2 (s, 5.32 ppm).

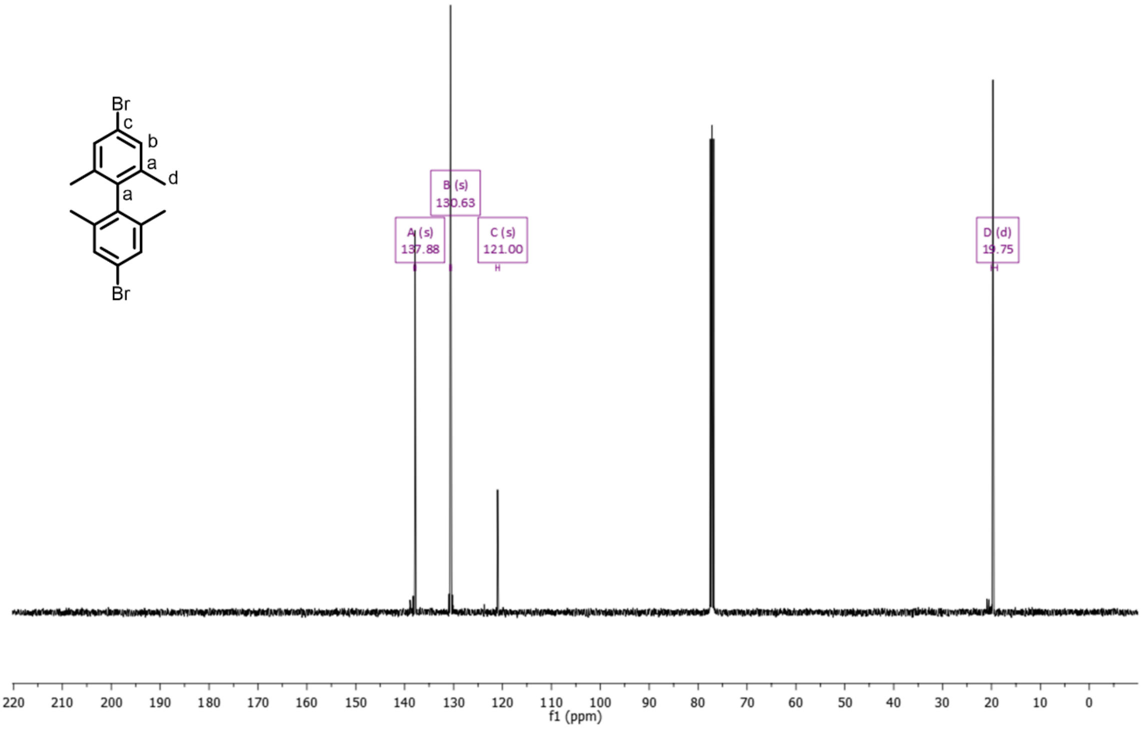

Figure S43. ^{13}C NMR spectrum of **BPDA-4-2Br** in $CDCl_3$.

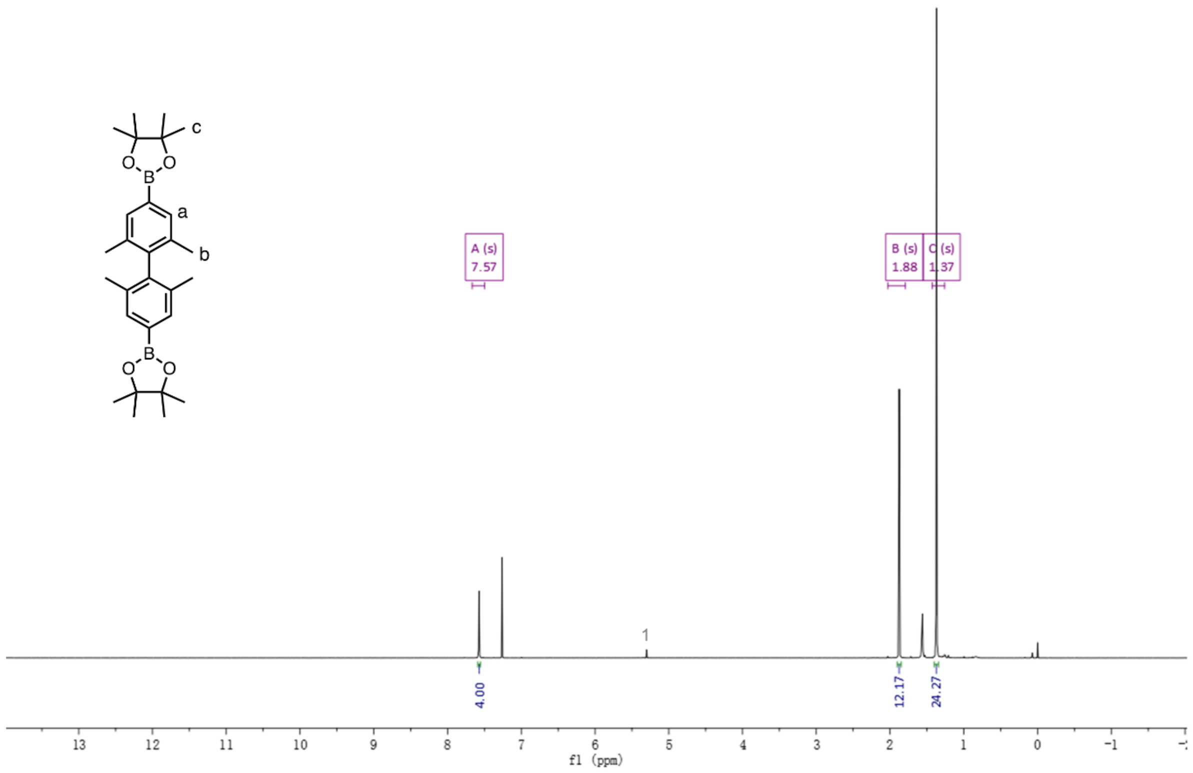

Figure S44. ^{1}H NMR spectrum of **BPDA-4-2Bpin** in $CDCl_3$. *1. CH_2Cl_2 (s, 5.30 ppm).

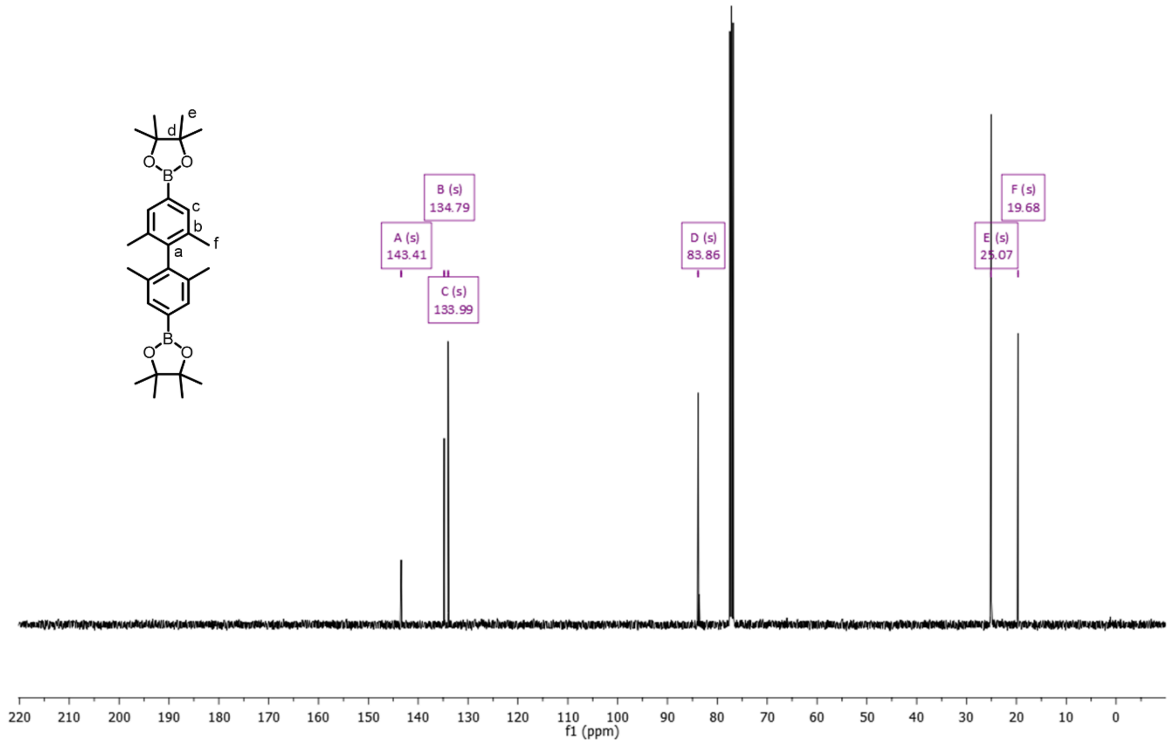

Figure S45. ^{13}C NMR spectrum of **BPDA-4-2Bpin** in $CDCl_3$. *C directly connected with B cannot be detected sometimes.

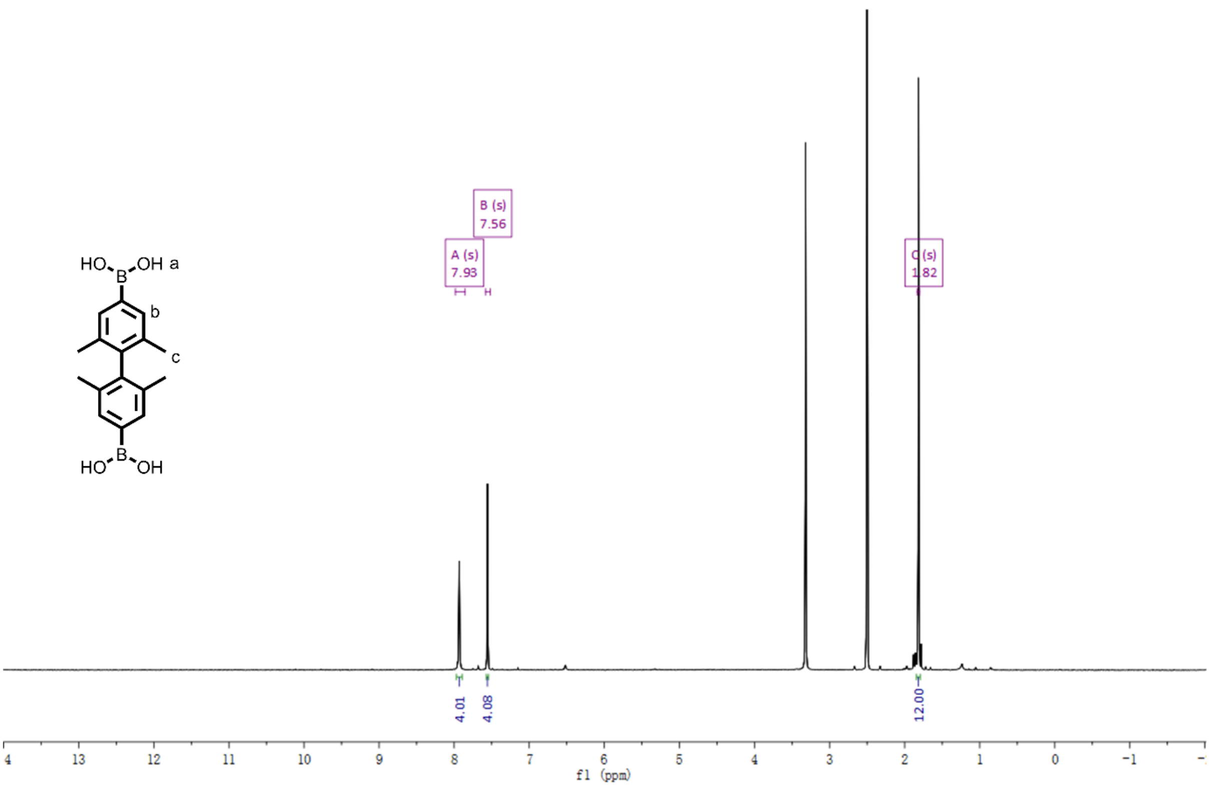

Figure S46. ^{1}H NMR spectrum of **BPDA-4** in dmso-d_6.

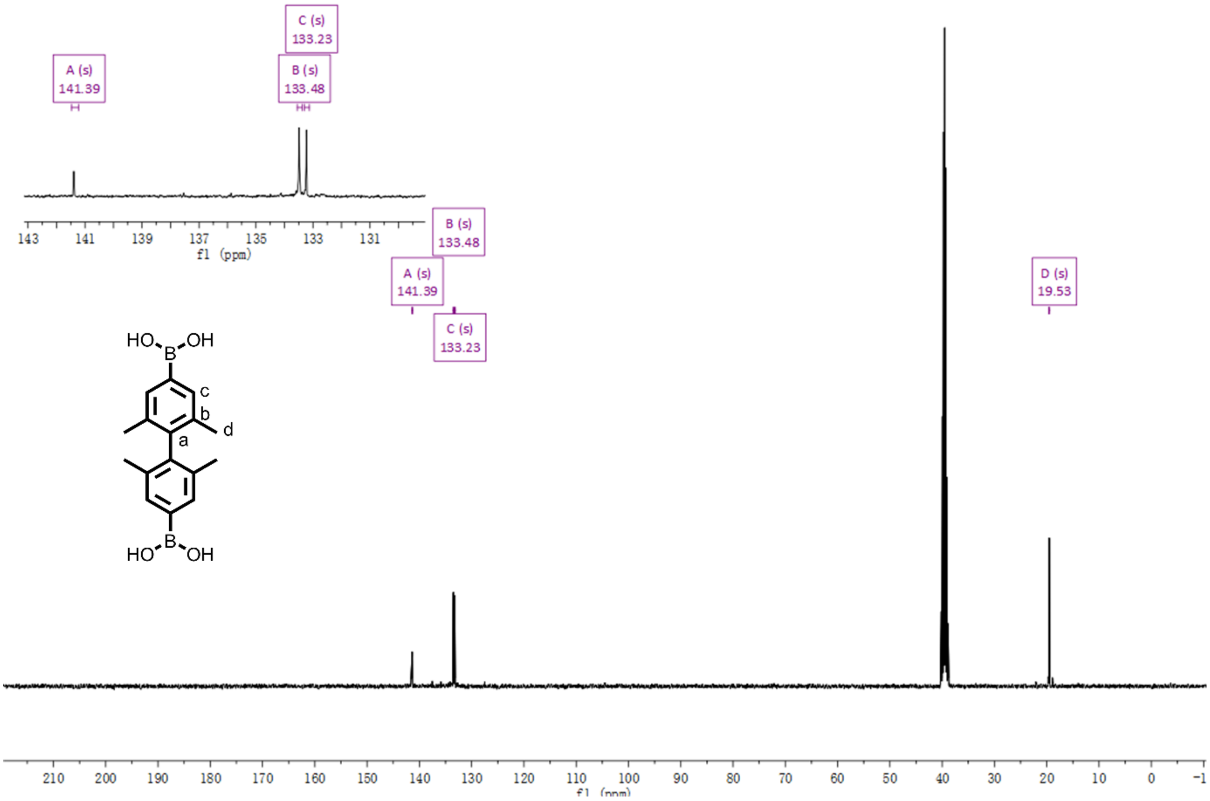

Figure S47. ^{13}C NMR spectrum of **BPDA-4** in dmso-d_6. *C directly connected with B cannot be detected sometimes.

14. Control study of $(OH)_8PcCo$ reacts with $B(OH)_3$ in DEF

The condensation between $(OH)_8PcCo$ with $B(OH)_3$ was conducted under the same condition as for the synthesis of BPDA-4-COF, but replacing linker BPDA-4 with $B(OH)_3$ (1.9 mg, 0.03 mmol). PXRD test was performed for the obtained COF.

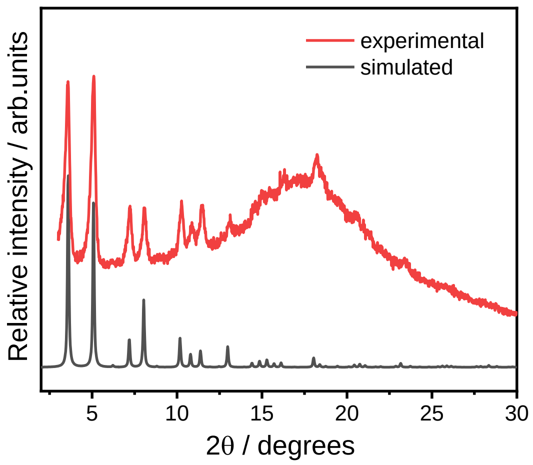

Figure S48. PXRD comparison between the obtained COF (from the condensation of $(OH)_8PcCo$ with $B(OH)_3$ in DEF) with the simulated pattern based-on the 3D spiroborate COF model. PXRD comparison result shows that the obtained COF is of 3D spiroborate structure.

*This result of the successful formation of **BPDA-4-COF** from the condensation between $(OH)_8PcCo$ with $B(OH)_3$ in DEF alone is not enough to support the mechanism of initial protodeboronation of boronic acid, because in the pathway of initial bornate ester formation followed by hydrolysis and the protodeboantion of boronic acid, the formation of the spiroborate structure is also based-on the reaction between $(OH)_8PcCo$ with $[B(OH)_4]^-$.

15. References

(1) Metz, J.; Schneider, O.; Hanack, M. Synthesis and properties of substituted (phthalocyaninato)-iron and -cobalt compounds and their pyridine adducts. *Inorg. Chem.* 1984, 23 (8), 1065-1071.

(2) Wang, X.; Bahri, M.; Fu, Z.; Little, M. A.; Liu, L.; Niu, H.; Browning, N. D.; Chong, S. Y.; Chen, L.; Ward, J. W.; et al. A Cubic 3D Covalent Organic Framework with nbo Topology. *J. Am. Chem. Soc.* 2021, 143 (37), 15011-15016.

(3) Sheldrick, G. Crystal structure refinement with SHELXL. *Acta Crystallogr. Sect. C* 2015, 71 (1), 3-8.

(4) Sheldrick, G. SHELXT - Integrated space-group and crystal-structure determination. *Acta Crystallogr. Sect. A* 2015, 71 (1), 3-8.

(5) Dolomanov, O. V.; Bourhis, L. J.; Gildea, R. J.; Howard, J. A. K.; Puschmann, H. OLEX2: a complete structure solution, refinement and analysis program. *J. Appl. Crystallogr.* 2009, 42 (2), 339-341.

(6) Helms, A.; Heiler, D.; McLendon, G. Electron transfer in bis-porphyrin donor-acceptor compounds with polyphenylene spacers shows a weak distance dependence. *J. Am. Chem. Soc.* 1992, 114 (15), 6227-6238.

(7) Yonekawa, I.; Mutoh, K.; Kobayashi, Y.; Abe, J. Structurally and electronically modulated spin interaction of transient biradicals in two photon-gated stepwise photochromism. *Photochem. Photobiol. Sci.* 2018, 17 (3), 290-301.

(8) Mullin, W. J.; Pawle, R. H.; Sharber, S. A.; Müller, P.; Thomas, S. W. Programmed twisting of phenylene–ethynylene linkages from aromatic stacking interactions. *J. Mater. Chem. C* 2019, 7 (5), 1198-1207.

(9) Karabacak, M.; Kose, E.; Atac, A.; Asiri, A. M.; Kurt, M. Monomeric and dimeric structures analysis and spectroscopic characterization of 3,5-difluorophenylboronic acid with experimental (FT-IR, FT-Raman, 1H and 13C NMR, UV) techniques and quantum chemical calculations. *J. Mol. Struct.* 2014, 1058, 79-96.

(10) Martin, R. L.; Haranczyk, M. Construction and Characterization of Structure Models of Crystalline Porous Polymers. *Cryst. Growth Des.* 2014, 14 (5), 2431-2440.

(11) Neti, V. S. P. K.; Wu, X.; Hosseini, M.; Bernal, R. A.; Deng, S.; Echegoyen, L. Synthesis of a phthalocyanine 2D covalent organic framework. *CrystEngComm* 2013, 15 (36), 7157-7160.

(12) Wang, X.; Fellowes, T.; Bahri, M.; Qu, H.; Li, B.; Niu, H.; Browning, N. D.; Zhang, W.; Ward, J. W.; Cooper, A. I. 2D to 3D Reconstruction of Boron-Linked Covalent–Organic Frameworks. *J. Am. Chem. Soc.* 2024, 146 (20), 14128-14135.
